# Influence of Casein kinase II inhibitor CX-4945 on BCL6-mediated apoptotic signaling in B-ALL in vitro and in vivo

**DOI:** 10.1186/s12885-020-6650-9

**Published:** 2020-03-04

**Authors:** Anna Richter, Sina Sender, Annemarie Lenz, Rico Schwarz, Burkhard Hinz, Gudrun Knuebel, Anett Sekora, Hugo Murua Escobar, Christian Junghanss, Catrin Roolf

**Affiliations:** 1 Department of Medicine, Clinic III – Hematology, Oncology, Palliative Medicine, Rostock University Medical Center, Ernst-Heydemann-Strasse 6, 18057 Rostock, Germany; 2 Institute of Pharmacology and Toxicology, Rostock University Medical Center, Schillingallee 70, 18057 Rostock, Germany

**Keywords:** B-ALL, Apoptosis, CK2, CX-4945, BCL6, BACH2, CDC42, AKT, Pharmakokinetic

## Abstract

**Background:**

Casein kinase II (CK2) is involved in multiple tumor-relevant signaling pathways affecting proliferation and apoptosis. CK2 is frequently upregulated in acute B-lymphoblastic leukemia (B-ALL) and can be targeted by the ATP-competitive CK2 inhibitor CX-4945. While reduced proliferation of tumor entities including B-ALL after CX-4945 incubation has been shown in vitro and in vivo, the detailed way of action is unknown. Here, we investigated the influence on the PI3K/AKT and apoptosis cascades in vivo and in vitro for further clarification.

**Methods:**

A B-ALL xenograft model in NSG mice was used to perform in vivo longitudinal bioluminescence imaging during six day CX-4945 treatment. CX-4945 serum levels were determined at various time points. Flow cytometry of bone marrow and spleen cells was performed to analyze CX-4945-induced effects on tumor cell proliferation and distribution in B-ALL engrafted mice. ALL cells were enriched and characterized by targeted RNA sequencing. In vitro, B-ALL cell lines SEM, RS4;11 and NALM-6 were incubated with CX-4945 and gene expression of apoptosis regulators *BCL6* and *BACH2* was determined.

**Results:**

In B-ALL-engrafted mice, overall tumor cell proliferation and distribution was not significantly influenced by CK2 inhibition. CX-4945 was detectable in serum during therapy and serum levels declined rapidly after cessation of CX-4945. While overall proliferation was not affected, early bone marrow and spleen blast frequencies seemed reduced after CK2 inhibition. Gene expression analyses revealed reduced expression of anti-apoptotic oncogene *BCL6* in bone marrow blasts of CX-4945-treated animals. Further, BCL6 protein expression decreased in B-ALL cell lines exposed to CX-4945 in vitro. Surprisingly, levels of BCL6 opponent and tumor suppressor BACH2 also declined after prolonged incubation. Simultaneously, increased phosphorylation of direct CK2 target and tumor initiator AKT was detected at respective time points, even in initially pAKT-negative cell line NALM-6.

**Conclusions:**

The CK2 inhibitor CX-4945 has limited clinical effects in an in vivo B-ALL xenograft model when applied as a single drug over a six day period. However, gene expression in B-ALL cells was altered and suggested effects on apoptosis via downregulation of BCL6. Unexpectedly, the BCL6 opponent BACH2 was also reduced. Interactions and regulation loops have to be further evaluated.

## Background

Casein kinase II (CK2) is a constitutively active, ubiquitously expressed serine/threonine kinase aberrantly activated in numerous solid and hematological tumors including acute B-lymphoblastic leukemia (B-ALL) [1]. CK2 phosphorylates a variety of target proteins with numerous functions involved in cell cycle regulation, cell growth, proliferation, transcription, translation and apoptosis. It thus influences pathways involved in tumorigenesis like PI3K/AKT, JAK/STAT and NFkB [2, 3]. CK2 acts via the inactivation of tumor suppressor genes *PTEN* and *IKZF1* (*Ikaros*) as well as stimulating proliferation and cellular growth in lymphoid malignancies including B-ALL [4]. Further, the CK2-mediated induction of apoptotic pathways as an additional mode of action has been studied in solid tumors and acute myeloid leukemia (AML) [5–8] but remains largely uninvestigated in B-ALL so far.

CX-4945 is a selective, ATP competitive CK2 inhibitor and currently under investigation in clinical studies for renal tumors, cholangiocarcinoma, basal cell carcinoma and medulloblastoma. Our group as well as others have previously shown that CX-4945 inhibits tumor cell proliferation and metabolic activity in vitro and in vivo for numerous neoplastic entities including B-ALL [1, 9, 10]. Several mechanisms have been discussed to identify the inhibitor’s anti-proliferative mode of action. These include intervention or modification of signaling pathways like PI3K/AKT, DNA repair response, angiogenesis, splicing regulation, stress-induced cell death or epigenetic modulation [10–16]. Nevertheless, it is still unknown how anti-leukemic effects are evoked in B-ALL. Induction of CK2-mediated apoptotic cascades or inhibition of anti-apoptotic pathways by CX-4945 might be mechanisms involved.

It has been demonstrated that incubation of B-ALL cells with CX-4945 induces apoptosis, possibly via increased cellular stress or inhibition of NFkB signaling [10, 11, 17]. So far it has not been investigated if in vitro effects are also present if CX-4945 is applied in vivo in B-ALL models. Further, little is known about which signaling molecules are involved in CX-4945-induced pro-apoptotic mechanisms and how those cascades are regulated during therapeutic approaches. We recently reported CX-4945-induced effects in B-ALL xenografts [10]. In this follow-up study, we investigate the early molecular mechanisms of CK2 inhibitor CX-4945 on B-ALL. We aim to explore whether and how apoptotic processes play a role for the anti-leukemic properties of CX-4945.

## Methods

### Animal studies

NOD scid gamma mice (NOD.Cg-*Prkdc*^*scid*^ *Il2rg*^*tm1Wjl*^/SzJ, NSG, Charles River Laboratories, Sulzfeld, Germany) were bred and housed under specific pathogen-free conditions with access to water and standard chow ad libitum. All experiments were carried out in a laboratory setting and no intervention was performed within the animal housing and breeding rooms. Only healthy female animals aged 8 to 14 weeks and 19.1–27.7 g weight were included in the experiments. Study group sizes were four animals per time point and intervention group. Experiments were approved by the review board of the federal state Mecklenburg-Vorpommern, Germany (reference number: LALLF MV/7221.3–1.1-002/15).

Study endpoints for all mice used are listed in Additional File 1: Table S1. Female mice were i.v. injected with 2.5 × 10^6^ SEM cells stably transduced with GFP and enhanced firefly luciferase (ffLuc). Transfection was performed using the pCDH-EF1-MCS-T2A-copGFP vector (System Biosciences, Mountain View, CA, USA) using EcoRI and BamHI as previously described [18]. SEM-GFP-ffluc cells were kindly provided by Prof. Irmela Jeremias (Helmholtz Center Munich, Germany). Lentivirus production and cell transduction were carried out as described before [19]. Tumor cell engraftment was evaluated 7 days after injection via bioluminescence imaging (BLI) using the NightOWL LB 983 in vivo Imaging System (Berthold Technologies, Bad Wildbach, Germany) and Indigo software (Berthold Technologies, version 1.04). For detection, animals were intraperitoneally injected with 4.5 mg D-Luciferin (GOLDbiotechnology, St. Louis, MO, USA), anesthetized with ketamine (75 mg/kg) and xylazine (5 mg/kg) and imaged in dorsal and ventral position (60 s exposure, 560 nm emission). Mice were randomized based on weight, age and tumor cell engraftment on d7. CX-4945 was dissolved in 0.9% saline containing 5% DMSO. Animals were simultaneously treated with either vehicle (isotonic saline supplemented with 5% DMSO) or 50 mg/kg i.p. CX-4945 twice daily d7–12 based on previous dose finding studies. BLI was performed at d7, d10, d13 and d15. At d10, d13 or d15 mice were anesthetized and euthanized by cervical dislocation. Leukemic blast frequency was analyzed in peripheral blood (PB), bone marrow (BM) and spleen by flow cytometry (GFP^+^) using FACSCalibur and CellQuest™ Pro software (BD, Heidelberg, Germany). Means and standard deviation of BLI and blast frequency values of all mice of a study group were calculated. Student’s t-test was performed to compare study groups and values < 0.05 were considered significant. Spleens, tibiae and femora were stored on ice in PBS (Biochrom, Berlin, Germany) containing 2% FCS (Biochrom) until tumor cell isolation.

### ALL cell enrichment from spleen and bone marrow

Briefly, spleens were passed through 100 μm cell strainers and incubated with erythrocyte lysis buffer (155 mM NH_4_Cl, 10 mM KHCO_3_, 0.1 mM EDTA). BM cells were isolated from tibiae and femora using PBS containing 2% FCS. Leukemic blast frequency was determined in BM and spleen cell populations by flow cytometry (GFP^+^) using FACSCalibur and CellQuest™ Pro software (BD). For enrichment of human ALL cells, mouse cells were depleted using the Mouse Cell Depletion Kit (Miltenyi Biotec, Bergisch Gladbach, Germany) for use with AutoMACS® (Miltenyi Biotec) according to the manufacturer’s guidelines.

### Mass spectrometric analysis of CX-4945 in mice sera

CX-4945 was determined by liquid coupled tandem mass spectrometry (LC-MS/MS) in the sera of treated mice to which the internal standard acridin orange was added prior to extraction. For this purpose, 10–250 μl serum was extracted by triple liquid-liquid extraction with ethyl acetate and n-hexane (1:3 V/V). The first extraction was only ethyl acetate and the solvent was evaporated under nitrogen. The residue was then reconstituted in 100 μl of a mixture of mobile phases A and B (65% A) and 80 μl were mass spectrometrically analyzed. Mass spectrometric analysis was performed on a Micromass Quattro Micro™ API mass spectrometer. A HPLC Shimadzu LC-20 AD was used to separate the samples. The separation was performed using a Multospher 120 C18 AQ column 125 × 2 mm, 5 μm particle size (CS-Chromatographie Service GmbH, Langerwehe, Germany) coupled with a guard column 20 × 3 mm, 5 μm particle size at a flow rate of 0.3 ml/min. The mobile phase A consisted of water with 0.2% formic acid and the mobile phase B consisted of acetonitrile with 0.2% formic acid. CX-4945 was ionized by electrospray ionization and analyzed in positive mode using multiple reaction monitoring. The transitions were *m/z* 350.04 (cone voltage 40 V) to *m/z* 223.1 with a collision energy of 12 V and *m/z* 314.1 with a collision energy of 24 V. The transitions *m/z* 223.1 was used as quantifier and *m/z* 314.1 as qualifier. For the internal standard the following transitions were used: *m/z* 266.11 (cone voltage 24 V) to *m/z* 234.1 with 44 V collision energy and *m/z* 250.1 with 24 V. A standard solution curve was linear in a range of 5–100 nM. The concentration of CX-4945 in the sera of mice was obtained by extrapolation of the measured concentration to 1 ml.

### Custom panel targeted RNA sequencing

RNA isolation of BM ALL cell fractions was performed using the AllPrep DNA/RNA/Protein Mini Kit (Qiagen, Hilden, Germany). Expression analyses were performed on the Ion Personal Genome Machine™ (PGM™) System (Ion Torrent™) (Thermo Fisher Scientific, Massachusetts, USA). For targeted RNA sequencing, an in-house custom panel was designed using Ion AmpliSeq Designer (Thermo Fisher Scientific), containing 179 genes involved in B-cell-receptor- and PI3K/AKT pathway signaling. Ion AmpliSeq RNA Libraries were prepared according to the manufacturer’s protocol (MAN0006735). In brief, RNA was quantified with Qubit RNA HS Assay Kit (Thermo Fisher Scientific) and Qubit 2.0 Fluorometer (Life Technologies, CA, USA) and transcribed into cDNA by SuperScript VILO cDNA Synthesis Kit (Thermo Fisher Scientific). cDNA targets were amplified, amplicons partially digested, ligated to the adapters and purified with the Ion AmpliSeq™ Library Kit 2.0 (Thermo Fisher Scientific) in the ProFlex PCR System (Thermo Fisher Scientific). The final librabries were quantified by Ion Library TaqMan Quantification Kit (Thermo Fisher Scientific) using the ViiA 7 Real-Time PCR System (Thermo Fisher Scientific). Following this, template preparation was carried out with the Ion PGM Hi-Q View OT2 Kit – 200 using the Ion One Touch 2 Instrument (Thermo Fisher Scientific) and enrichment of template-positive Ion Sphere Particles (ISP) with the Ion OneTouch ES (Thermo Fisher Scientific). The sequencing reaction run was performed with the PGM System and 400 flows. The evaluation of data sets was performed using Transcriptome Analysis Console (TAC) Software 4.0.0.25 (Thermo Fisher Scientific). The following filter criteria were set to identify relevantly regulated genes: Avg (log2) > 5, fold change > 2 or < − 2, *p*-value < 0.05.

### Cell culture and inhibitor exposition

Human B-ALL precursor cell lines SEM (ACC 546), RS4;11 (ACC 508) and NALM-6 (ACC 128) were purchased from German Collection of Microorganisms and Cell Cultures (DSMZ, Braunschweig, Germany) in 2004 (SEM and RS4;11) and 2016 (NALM-6), cultured as recommended by the manufacturer at 37 °C and 5% CO_2_. Media were supplemented with 10% heat-inactivated FCS (Biochrom, Berlin, Germany) and 100 μg/ml penicillin and streptomycin (Biochrom, Berlin, Germany). All cell lines were regularly tested for mycoplasma contamination (PCR) and authenticity (cell surface markers by flow cytometry) shortly before experiment initiation. 3.3 × 10^5^ cells per ml were incubated with 5 μM CX-4945 (Hycultec, Beutelsbach, Germany) or DMSO (control) for up to 96 h. Experiments were carried out in biological triplicates.

### Western blot

Cells were lysed using RIPA buffer (Cell Signaling) and ultra sound exposure. Proteins (30 μg) were separated on Midi gels (Bio-Rad, Munich, Germany), blotted onto a PVDF membrane (Bio-Rad) using the Trans-Blot® Turbo™ Transfer System (Bio-Rad, 2.5 A, 25 V, 10 min), blocked in LI-COR (Lincoln, NE, USA) blocking buffer and detected via LI-COR Odyssey Imaging System and Image Studio Lite software. The following antibodies were used for protein detection: pAKT (Ser473), AKT, BCL6 (clone D4I2V), BACH2 (clone D3T3G; all Cell Signaling), GAPDH (clone ZG003; Invitrogen), IRDye® 680RD Goat anti-Mouse, IRDye® 800CW Goat anti-Mouse, IRDye® 680RD Goat anti-Rabbit and IRDye® 800CW Goat anti-Rabbit (all LI-COR). Experiments were carried out in biological triplicates.

### *BCL6* and *BACH2* gene expression analysis

RNA isolation of BM tumor cell fractions and cell culture samples was performed using the AllPrep DNA/RNA/Protein Mini Kit (Qiagen, Hilden, Germany) and miRNeasy® Mini Kit (Qiagen), respectively, and followed by cDNA synthesis using the PrimeScript™ RT Reagent Kit (Takara Bio Europe, Saint-Germain-en-Laye, France) according to the manufacturers’ protocols. Gene expression analysis was carried out in technical triplicates using TB Green™ Premix Ex Taq™ II mastermix (Takara Bio Europe) with 0.3 μM primers (*BCL6* forward CTGTGATGGCCACGGCTAT; *BCL6* reverse CGGCAAGTGTCCACAA; *BACH2* forward GCGGCCCCAAATTAAATGT; *BACH2* reverse AACGATCCGGATTCGTCACT; *GAPDH* forward CTGCACCACCAACTGCTTAG; *GAPDH* reverse GTCTTCTGGGTGGCAGTGAT) and 10 ng cDNA in a ViiA7 Real Time PCR system (Applied Biosystems, Foster City, CA, USA) using the following protocol: 15 min initial denaturation at 95 °C followed by 45 cycles of 15 s denaturation at 94 °C, 30 s annealing at 54 °C and 30 s elongation at 72 °C. Relative gene expression was normalized to *GAPDH* and calculated using the 2^-ΔΔCt^ formula. Cell culture experiments were carried out in biological triplicates. Four mice per group and time point were analyzed. Results are described as mean ± standard deviation. Significance (*p* < 0.05) was estimated by 2-tailed student’s t test.

## Results

### Early effects of targeted CK2 inhibition on B-ALL xenograft mice

**Fig. 1:**
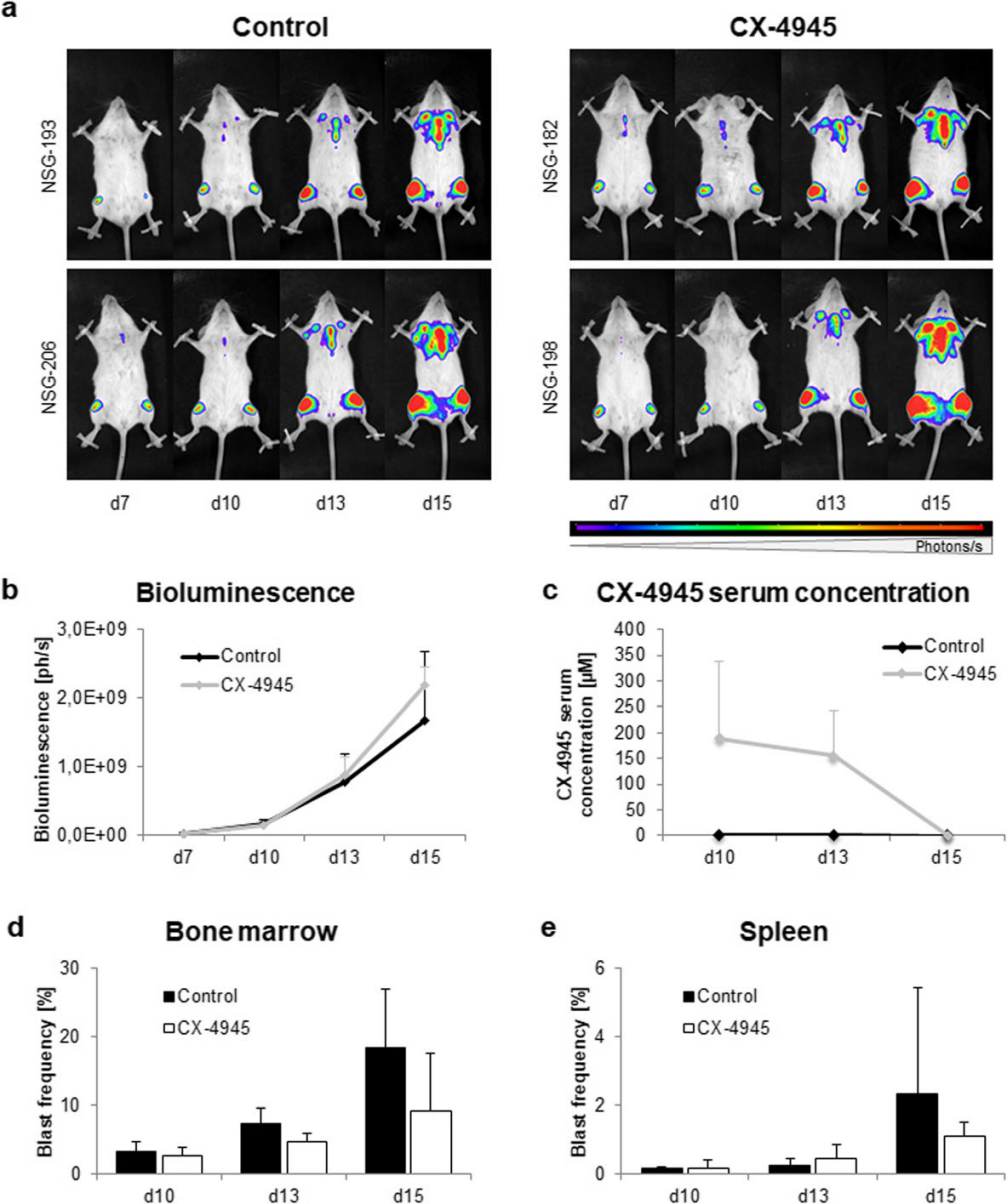
Evaluation of CX-4945 application on NSG mice engrafted with SEM B-ALL cells. NSG mice were i.v.-injected with 2.5 × 10^6^ GFP- and luciferase-transduced SEM cells and treated with vehicle (control) or 50 mg/kg CX-4945 i.p. twice daily from d7–13. Mice were sacrificed on d10, d13 or d15 for subsequent analyses. **a** Representative images of longitudinal bioluminescence imaging of two control animals and two CX-4945-treated animals in dorsal position. **b** Longitudinal bioluminescence imaging was conducted at d7 to verify tumor cell engraftment and subsequently repeated at all study days. Increasing luminescence is proportional to proliferation of luciferase-expressing blasts. Quantification of full body bioluminescence (ph/s) after treatment was performed by adding total luminescence signals of dorsal and ventral imaging. Four animals per time point and study group; mean ± standard deviation. **c** Pharmacokinetic analyses were conducted at d10, d13 and d15 to investigate CX-4945 serum concentrations. Serum levels were determined by liquid coupled tandem mass spectrometry. Four animals per time point and study group; mean ± standard deviation. **d,e** Influence of CX-4945 on blast frequency in bone marrow **d** and spleen **e**. Tumor cell frequency was evaluated by flow cytometry of GFP^+^ leukemic blasts. Four animals per time point and study group; mean ± standard deviation

Previous studies involving CX-4945 anti-tumor regimens are mainly based on observations made at time points several days after the last therapeutic dose. We herein focused on CX-4945-mediated effects during and shortly after therapy. To evaluate the immediate effect of CK2 inhibition (twice daily from d7 to d13) on the proliferation of SEM cells in NSG mice, animals were sacrificed during (d10), immediately after (d13) or 48 h after the last therapeutic dose (d15). To examine tumor cell proliferation and distribution, longitudinal bioluminescence imaging (BLI) of all animals was performed on d7, d10, d13 and d15. No differences in blast distribution (Fig. 1a) and proliferation (Fig. 1b) were observed between treated and untreated animals.

Pharmacokinetic experiments were conducted to elucidate in which concentrations CX-4945 was present in serum of treated mice (Fig. 1c). During therapy CX-4945 concentrations of 190 ± 150 μM (d10) and 155 ± 88 μM (d13) were measured. CX-4945 levels then declined rapidly: two days after the final CX-4945 application (d15) traces were detectable only in one out of four mice. No traces of CX-4945 were detected in untreated animals.

Investigations of cells and tissues revealed only minimal changes. BM tumor cell frequency determined by flow cytometry was lower in CX-4945-treated animals compared to controls at all time points (Fig. 1d). Tumor cell frequency in control animals increased rapidly from 3.3 ± 1.4% (d10) to 18.4 ± 8.5% (d15) while CX-4945-treated mice showed decelerated blast counts (2.7 ± 1.2%, 4.7 ± 1.3%, 9.2 ± 8.5% at d10, d13, d15, respectively). Spleen infiltration of ALL cells was reduced accordingly (Fig. 1e; Control: 2.3 ± 3.1% vs CX-4945: 1.1 ± 0.4% at d15).

Further, BLI was performed for several organs to identify minor tumor cell infiltration (Additional File 2: Fig. S1). Blasts were mostly localized in tibiae, femora and skull while infiltration of sternum, spleen, liver and lung was rarely observed. Tumor cell distribution was comparable in controls and CX-4945-treated animals.

### Downregulation of *BCL6* induces apoptosis in BM-derived ALL blasts

**Fig. 2:**
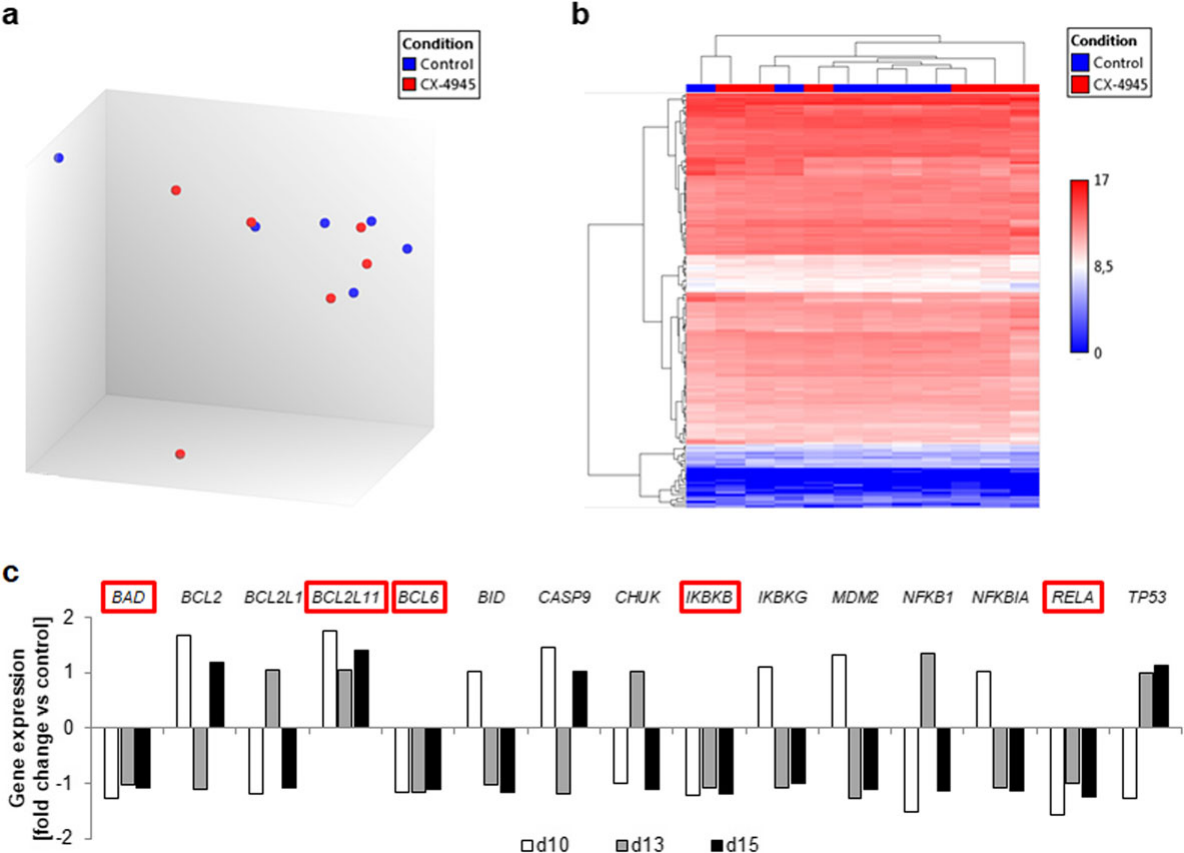
Custom panel targeted RNA sequencing of treated and untreated mice. Bone marrow-derived leukemic blast populations of controls and treated mice sacrificed at d10, d13 and d15 (two mice per group and time point) were analyzed for changes in gene expression of 177 genes involved in BCR, PI3K/AKT and CK2 downstream signaling. **a** Principal component analysis blot of control animals (blue) and CX-4945-treated animals (red) demonstrating no distinct clustering of subgroups. **b** Hierarchical clustering of all genes and all animals throughout all observation time points. **c** Fold changes of apoptosis-related genes within the panel after CX-4945 treatment compared to time-matched controls. No significant regulation (fold change > 2 or < − 2) was observed. Red boxes indicate genes with consistent gene expression changes throughout all time points

To identify molecular pathways involved in the decreased BM and spleen blast infiltration, we next performed gene expression profiling. We analyzed 177 genes involved in B cell receptor (BCR), PI3K/AKT and CK2 downstream signaling in selected bone marrow blast samples (Additional File 1: Table S2). Samples taken at the distinct observation time points (d10, d13, d15) were analyzed separately, revealing significant upregulation of *CDC42*, *CD19* and *JAK1* after CX-4945 therapy at d10 (Fold changes 2.22, 2.71 and 2.29 compared to controls, respectively). However, these changes were not found at d13 and d15. In fact, no relevant up- or downregulation was present at the latter time points. Comparing all controls and treated animals, no relevant difference was observed and groups did not form distinct clusters (Fig. 2a, b).

Throughout all time points, gene expression of anti-apoptotic transcription factor *BCL6* was slightly reduced after CX-4945 therapy (Fold changes − 1.17, − 1.15 and − 1.11 compared to controls at d10, d13 and d15, respectively). In contrast, expression of *BCL2L11*, an inducer of apoptosis, was increased (Fold changes 1.76, 1.04, 1.40; Additional File 3: Table S2), possibly encouraging pro-apoptotic signaling (Fig. 2c). Interestingly, apoptosis-inducing *BAD*, *IKBKB* and *RELA* expression was decreased, suggesting the previously observed induction of cell death [10] was probably not mediated through NFkB. According to wikipathways, 15 genes included in our panel are involved in apoptotic signaling [20]. None of these was significantly regulated by CX-4945 therapy at any time point. Most genes showed ambiguous gene expression profiles during observation time points. Raw data, fold changes and *p*-values of all genes for respective time points are summarized in Additional File 4: Table S3.

### CX-4945-induced apoptosis is evoked by BCL6 deregulation

As *BCL6* was found consistently downregulated in RNA sequencing analyses and can be regulated by CX-4945 target Ikaros, we evaluated this pathway in more detail. We also investigated BCL6 counterpart and tumor suppressor BACH2.

For characterization of BCL6 and BACH2 involvement in anti-proliferative effects after CK2 inhibition, MLL-rearranged pro-B-ALL cell lines SEM and RS4;11 as well as BCR^+^ pre-B-ALL cell line NALM-6 were incubated with CX-4945 for up to 96 h. All cell lines initially expressed different amounts of BACH2 and BCL6 proteins (Additional File 5: Fig. S2). High levels of BCL6 correlated with low BACH2 expression (SEM, RS4;11) and vice versa (NALM-6).

**Fig. 3:**
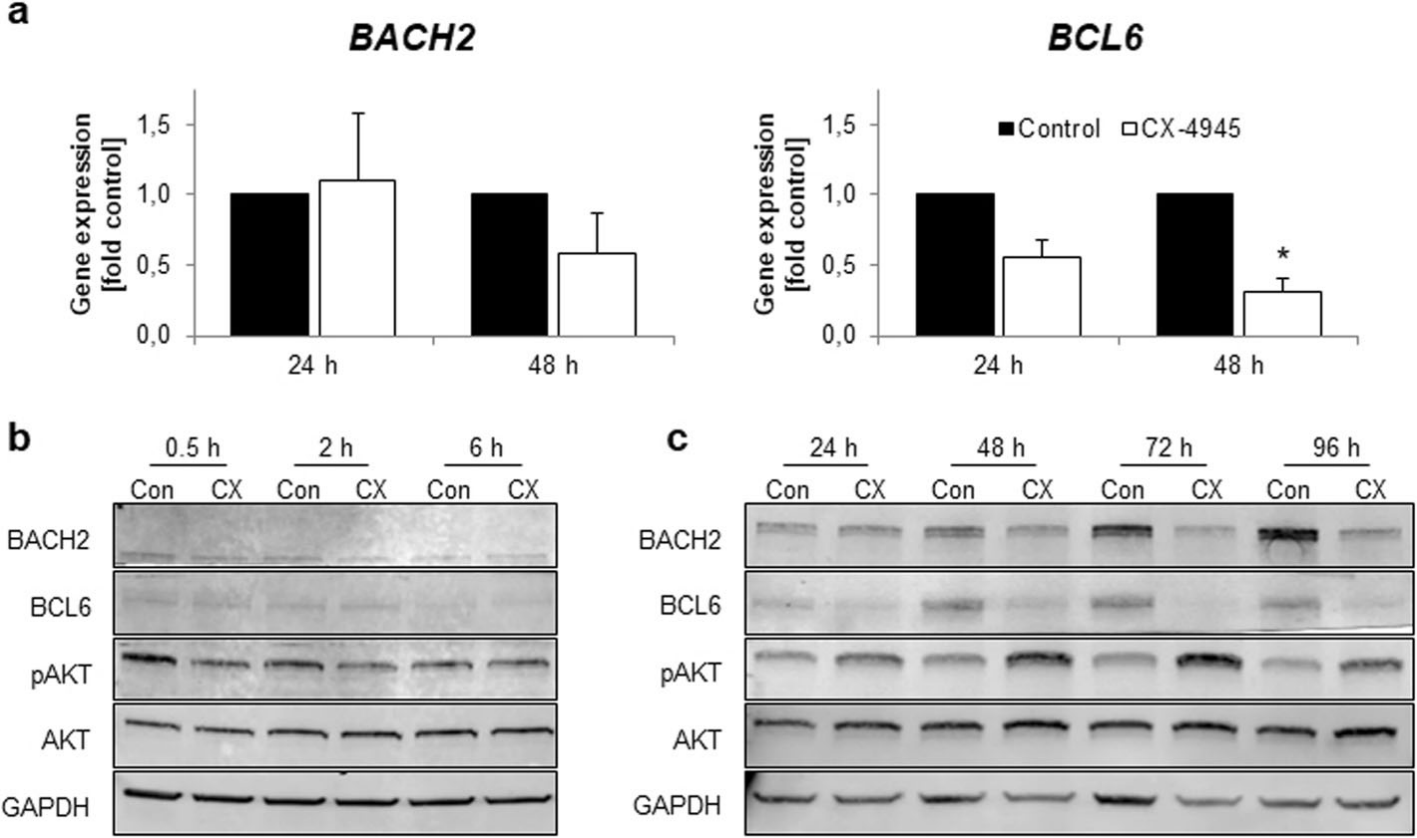
Evaluation of CX-4945-induced effects on BACH2 and BCL6 gene and protein expression in SEM cells. SEM cells were cultured and incubated with 5 μM CX-4945 or DMSO (control) for up to 96 h. **a** Changes in *BACH2* and *BCL6* gene expression were assessed by qRT-PCR after 48 h and 72 h. Mean values of ΔCT values from DMSO-treated control cells were calculated and set to 1 for each time point. ΔΔCT values were calculated for CX-4945-treated samples and compared to the respective time-matched control. Analyses were carried out in three independent biological and technical replicates. Mean ± standard deviation; * *p* < 0.05. **b, c** Analysis of BACH2 and BCL6 protein expression as well as AKT phosphorylation was carried out by western blot with GAPDH as housekeeping gene. Representative images of three independent biological experiments. Blots were processed and cropped using Image Studio Lite 5.2 software and MS PowerPoint (2011) to improve clarity and conciseness. Full size blots are uploaded in Additional File 8: Fig. S5. **b** Short term effects of CX-4945 incubation were determined after 0.5 h, 2 h and 6 h. **c** Long term effects of CX-4945 incubation were determined after 24 h, 48 h, 72 h and 96 h

In SEM cells, *BACH2* gene expression (Fig. 3a) and protein expression (Fig. 3b, c) remained constant during short term-incubation (< 24 h) while incubation for extended periods induced decreased gene and protein expression. Similar results were observed for *BCL6* with significantly reduced gene expression levels after 48 h (0.31-fold, *p* = 0.011; 24 h: 0.55-fold, *p* = 0.053). BCL6 protein levels also decreased after 24 h, 48 h, 72 h and 96 h of CX-4945 incubation.

Phosphorylation of AKT residue Ser473 was evaluated to further characterize the effect of CX-4945 on PI3K signaling. AKT phosphorylation decreased after short term incubation with CX-4945 (Fig. 3b). Conversely, pAKT levels were elevated after extended incubation periods (Fig. 3c). Total AKT protein expression remained constant throughout all time points observed.

To further validate the observed effects, the influence of CX-4945 on B-ALL cell lines of distinct molecular backgrounds (RS4;11, NALM-6) was determined on protein level. Both cell lines exhibited reduced BACH2 and BCL6 expression detectable from as early as 6 h incubation (Additional File 6: Fig. S3). In RS4;11 cells, pAKT levels decreased during short term incubation (Additional File 6: Fig. S3a) before increasing after 48 h, 72 h and 96 h CX-4945 incubation compared to DMSO-treated control cells (Additional File 6: Fig. S3b). Even though no basal AKT phosphorylation was detected in NALM-6 cells, pAKT levels increased after extended incubation with CX-4945 (Additional File 6: Fig. S3d).

For comparison with *BCL6* and in vitro data, gene expression analysis of BM-derived tumor cell populations was subsequently performed for *BACH2* (Additional File 7: Fig. S4). *BACH2* gene expression was slightly increased at d10 and d15 (1.19-fold and 1.26-fold compared to control animals, respectively). Though, changes were not significant (*p* = 0.234 and 0.333, respectively), matching our findings that changes in *BCL6* rather than *BACH2* gene expression contribute to altered apoptotic signaling.

## Discussion

CK2 is frequently upregulated in many neoplasms including B-ALL [4]. The selective CK2 inhibitor CX-4945 has previously been shown to exhibit anti-proliferative effects in vitro and heterogeneously in vivo [10, 11, 15, 17, 21–23]. However, molecular mechanisms and the exact mode of action of CX-4945 in B-ALL remain widely unknown. We have previously demonstrated induction of apoptosis in B-ALL cell lines including SEM [10]. Other groups also described CX-4945-mediated apoptosis in various tumor cells including B-ALL [8, 11, 13, 15, 17, 22, 24–27]. Thus, the aim of the current study was to evaluate whether CX-4945 treatment of B-ALL xenograft mice results in early reduced proliferation. Further, we investigated CX-4945-induced apoptotic and molecular processes during and after CK2 inhibition using in vitro and in vivo approaches. Our results suggest that downregulation of the oncogenic transcription factor *BCL6* might contribute to anti-proliferative signaling.

While previous in vitro experiments using CX-4945 demonstrated significant anti-proliferative effects in B-ALL cell lines, our results could not demonstrate this effect in a B-ALL xenograft model. In contrast, Song et al. detected prolonged survival and reduced tumor cell proliferation in B-ALL engrafted mice [28]. This discrepancy might be due to different cell lines used or varying therapeutic regimens. Our study used lower CX-4945 doses and shorter application periods than Song et al. Also, we evaluated tumor cell proliferation during or immediately after our short therapeutic period, explaining the lack of anti-proliferative action. In line with our observations, Prins et al. reported only minimal effects of CX-4945 on de novo B-ALL xenograft mice [22]. To ensure that the lack of anti-proliferative effects was not due to limited bioavailability, we performed pharmacokinetic studies and demonstrated that CX-4945 was present in blood serum samples of treated animals. Achieved CX-4945 concentrations are similar to otherwise reported effective concentrations. Serum levels declined rapidly when the drug was removed, which is in line with the observations of Siddiqui-Jain et al. who also reported a quick reduction of plasma concentrations from as early as 15 min after CX-4945 removal [13]. Other groups calculated a CX-4945 half-life of ~ 5 h in mice [29]. This rapid decline in CX-4945 bioavailability might explain the continued proliferation of B-ALL tumor cells after therapy we observed in our previous study. This finding suggests that prolonged or continuous CX-4945 exposure might overcome the limited therapeutic potential [10].

Still, in our present cohort CX-4945 treatment of mice engrafted with B-ALL cell line SEM resulted in decreased blast frequencies in BM and spleen. This indicates that even short application periods and a moderate dosis of CX-4945 are sufficient to induce signaling-modulating changes in tumor cells. Focusing on BCR- and PI3K/AKT-related genes, we performed targeted RNA sequencing to firstly evaluate if PI3K downstream signaling modifications could be responsible for the observed effects. Analysis of BM-derived blasts revealed a significant upregulation of *CDC42*, *CD19* and *JAK1* after CX-4945 treatment. *JAK1* activation and *CD19* overexpression are generally not associated with anti-leukemic signaling but with leukemogenesis [30, 31]. In contrast, the small GTPase CDC42 plays a key role in cell cycle regulation [32] and might be involved in apoptotic signaling via activation of JNK and FasL in HL-60 and Jurkat cells [33, 34].

We then evaluated the gene expression of apoptosis-related genes after CX-4945 incubation in more detail. We found that the anti-apoptotic transcription factor *BCL6* was constantly, yet not significantly, downregulated throughout all observation time points. Most other genes involved in apoptosis showed ambiguous regulation patterns. In line with this finding, Ge et al. recently reported that in vitro *BCL6* gene expression is regulated by direct CX-4945 target Ikaros, and that increased *BCL6* expression in adult B-ALL patients is associated with inferior outcomes [35]. We subsequently evaluated protein expression of BCL6 and its opponent, tumor-suppressing transcription factor BACH2 in B-ALL cell lines after CX-4945 incubation and found reduced BCL6 levels. Unexpectedly and in contrast to Ge et al., BACH2 gene and protein levels were also decreased. This could be explained by CX-4945-mediated regulatory mechanisms other than Ikaros binding, or by upstream signaling modulation in the cell lines used. *BACH2* gene expression can be controlled by hypoxia-induced factor 1α, a target protein of CK2 [36–38]. Also, Tamahara et al. further demonstrated AKT- and mTOR-mediated inhibition of gene and protein expression of BACH2 [39]. This matches our findings of both decreased BACH2 levels as well as AKT activation occurring after extended CX-4945 incubation periods. This observation suggests the possibility of a shared AKT and BACH2 regulatory mechanism.

In vitro western blot analyses in SEM and RS4;11 cells demonstrated that the direct CK2 target protein AKT was initially dephosphorylated by CK2 inhibition. This results in decreased PI3K/AKT pathway activity and potential induction of apoptotic signaling via downstream mechanisms. Unexpectedly, further CX-4945 incubation evoked strong phosphorylation of AKT even in the initially phospho-AKT-negative cell line NALM-6, suggesting an induction of cellular escape mechanisms to evade apoptosis. The analyzed AKT residue Ser473 is phosphorylated by PI3K/AKT pathway member mTOR and necessary for AKT kinase activity, underlining the involvement of this signaling cascade in CX-4945-mediated anti-leukemic effects [40]. Recently Chen et al. as well as Baumgarten et al. both reported feedback loops between AKT and MEK signaling, suggesting increased AKT phosphorylation might be evoked by escape strategies involving upregulation of the MEK pathway [41, 42]. This also fits in well with the increased gene expression of *CDC42* observed after CX-4945 treatment, with CDC42 being involved in JNK and p38 signaling cascades [43]. However, whole transcriptome and whole methylome sequencing approaches as well as further pharmacokinetic experiments are necessary to elucidate in detail how CX-4945 influences distinct signaling cascades. In addition, combinatory approaches using mTOR, AKT and MEK inhibitors can shed light on so far unexplored pathway regulation and feedback loops. Also, these results should be validated in human primary B-ALL cells.

## Conclusions

In conclusion, we herein identify a potential mechanism for the regulation of apoptotic processes in B-ALL in vitro and in vivo. Apoptosis is probably evoked by Ikaros-mediated downregulation of BCL6 and tightly regulated by AKT. Limitations in anti-proliferative and pro-apoptotic CX-4945-induced effects could possibly be overcome by additional application of AKT or MEK inhibitors.

## Supplementary information

**Additional File 1: Table S1.** List of all mice recruited in the study, respective end points and analyses conducted.

**Additional File 2: Figure S1.** Evaluation of CX-4945 application on organ infiltration in SEM-engrafted NSG mice. NSG mice were i.v.-injected with 2.5 × 10^6^ GFP- and luciferase-transduced SEM cells and treated with vehicle (control) or 50 mg/kg CX-4945 i.p. twice daily from d7–13. Mice were sacrificed on d10, d13 or d15 for subsequent analyses. Bioluminescence imaging of brain, skull, lung, heart, liver, sternum, spleen, kidney, femur, tibia and fat tissue was performed directly after mice were sacrificed. Four animals per time point and study group.

**Additional File 3: Table S2.** Gene expression fold changes of genes involved in apoptotic processes.

**Additional File 4: Table S3.** Gene expression fold changes and *p*-values of all genes included in the BCR/PI3K/CK2 panel.

**Additional File 5: Figure S2.** Basal characterization of protein expression in B-ALL cell lines SEM, RS4;11 and NALM-6. Analysis of BACH2 and BCL6 protein expression was carried out by western blot with GAPDH as housekeeping gene. Representative images of three independent biological experiments. Blots were processed and cropped using Image Studio Lite 5.2 software and MS PowerPoint (2011) to improve clarity and conciseness. Full size blots are uploaded in Additional File 8: Fig. S5.

**Additional File 6: Figure S3.** Evaluation of AKT phosphorylation, BACH2 and BCL6 protein expression in RS4;11 and NALM-6 cells. RS4;11 (**a, b**) and NALM-6 cells (**c, d**) were cultured and incubated with 5 μM CX-4945 or DMSO (control) for up to 96 h. Analysis of BACH2 and BCL6 protein expression as well as AKT phosphorylation was carried out by western blot with GAPDH as housekeeping gene. Representative images of three independent biological experiments. Short term effects of CX-4945 incubation were determined after 0.5 h, 2 h and 6 h (**a, c**). Long term effects of CX-4945 incubation were determined after 24Blots were processed and cropped using Image Studio Lite 5.2 software and MS PowerPoint (2011) to improve clarity and conciseness. Full size blots are uploaded in Additional File 8: Fig. S5.

**Additional File 7: Figure S4.** Gene expression analysis of *BACH2*. Bone marrow-derived leukemic blast populations of controls and treated mice sacrificed at d10, d13 and d15 were analyzed for changes in gene expression of *BACH2* using qPCR. Mean values of ΔCT values from controls were calculated and set to 1 for each time point. ΔΔCT values were calculated for CX-4945-treated samples and compared to the respective time-matched control. Analyses were carried out in three technical replicates. Four animals per time point and study group; mean ± standard deviation.

**Additional File 8: Figure S5.** Original blots for Fig. 3b, c, S2, S3. Red boxes indicate the regions used in the respective Figures.

## Data Availability

The datasets used and/or analysed during the current study are available from the corresponding author on reasonable request.

## Abbreviations

AML: Acute myeloid leukemia
B-ALL: Acute B-lymphoblastic leukemia
BCR: B cell receptor
BLI: Bioluminescence imaging
BM: Bone marrow
CK2: Casein kinase II
Ffluc: Firefly luciferase
LC-MS/MS: Liquid phase coupled tandem mass spectrometry
PB: Peripheral blood
