# Supplementary material for: Influence of Casein kinase II inhibitor CX-4945 on BCL6-mediated apoptotic signaling in B-ALL in vitro and in vivo
Source: BMC Cancer. 2020 Mar 4;20:184. doi: 10.1186/s12885-020-6650-9 (PMC7057698; doi:10.1186/s12885-020-6650-9)
Supplement: Supplementary file 1 — Additional File 1: Table S1. List of all mice recruited in the study, respective end points and analyses conducted. [file 12885_2020_6650_MOESM1_ESM.docx]

Table S1: List of all mice recruited in the study, respective end points and analyses conducted.

| Controls | | | | | | | CX-4945-treated | | | | | | |
| --- | --- | --- | --- | --- | --- | --- | --- | --- | --- | --- | --- | --- | --- |
| Mouse ID | End point | Flow cytometry | Biolumines-cence | Gene expression | RNA panel seq | Pharmaco-kinetic | Mouse ID | End point | Flow cytometry | Biolumines-cence | Gene expression | RNA panel seq | Pharmaco-kinetic |
| NSG-180 | d10 | √ | √ | √ |  | √ | NSG-178 | d10 | √ | √ | √ |  | √ |
| NSG-183 | d10 | √ | √ | √ |  | √ | NSG-186 | d10 | √ | √ | √ |  | √ |
| NSG-203 | d10 | √ | √ | √ | √ | √ | NSG-199 | d10 | √ | √ | √ | √ | √ |
| NSG-204 | d10 | √ | √ | √ | √ | √ | NSG-200 | d10 | √ | √ | √ | √ | √ |
| NSG-176 | d13 | √ | √ | √ |  | √ | NSG-184 | d13 | √ | √ | √ | √ | √ |
| NSG-179 | d13 | √ | √ | √ |  | √ | NSG-187 | d13 | √ | √ | √ |  | √ |
| NSG-202 | d13 | √ | √ | √ | √ | √ | NSG-195 | d13 | √ | √ | √ |  | √ |
| NSG-205 | d13 | √ | √ | √ | √ | √ | NSG-201 | d13 | √ | √ | √ | √ | √ |
| NSG-181 | d15 | √ | √ | √ |  | √ | NSG-182 | d15 | √ | √ | √ |  | √ |
| NSG-193 | d15 | √ | √ | √ | √ | √ | NSG-185 | d15 | √ | √ | √ |  | √ |
| NSG-196 | d15 | √ | √ | √ |  | √ | NSG-197 | d15 | √ | √ | √ | √ | √ |
| NSG-206 | d15 | √ | √ | √ | √ | √ | NSG-198 | d15 | √ | √ | √ | √ | √ |
