# Supplementary figures and images for: Influence of Casein kinase II inhibitor CX-4945 on BCL6-mediated apoptotic signaling in B-ALL in vitro and in vivo
Source: BMC Cancer. 2020 Mar 4;20:184. doi: 10.1186/s12885-020-6650-9 (PMC7057698; doi:10.1186/s12885-020-6650-9)

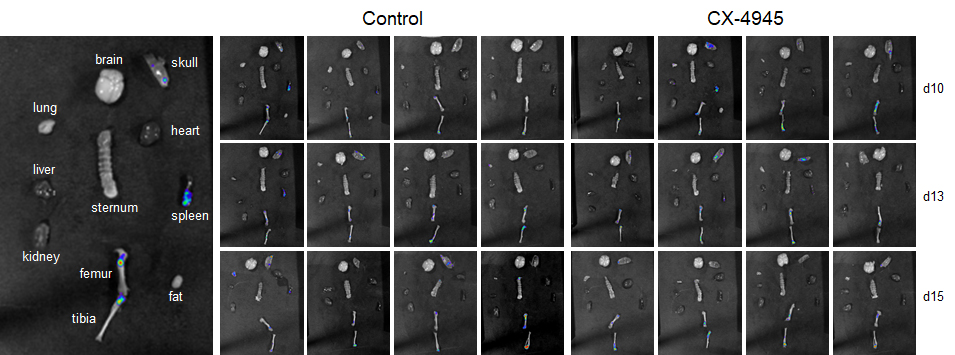

Supplement: Supplementary file 2 — Additional File 2: Figure S1. Evaluation of CX-4945 application on organ infiltration in SEM-engrafted NSG mice. NSG mice were i.v.-injected with 2.5 × 10^6^ GFP- and luciferase-transduced SEM cells and treated with vehicle (control) or 50 mg/kg CX-4945 i.p. twice daily from d7–13. Mice were sacrificed on d10, d13 or d15 for subsequent analyses. Bioluminescence imaging of brain, skull, lung, heart, liver, sternum, spleen, kidney, femur, tibia and fat tissue was performed directly after mice were sacrificed. Four animals per time point and study group. [file 12885_2020_6650_MOESM2_ESM.jpg]

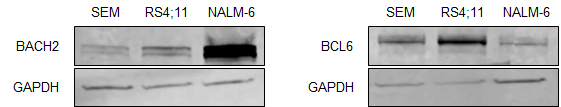

Supplement: Supplementary file 5 — Additional File 5: Figure S2. Basal characterization of protein expression in B-ALL cell lines SEM, RS4;11 and NALM-6. Analysis of BACH2 and BCL6 protein expression was carried out by western blot with GAPDH as housekeeping gene. Representative images of three independent biological experiments. Blots were processed and cropped using Image Studio Lite 5.2 software and MS PowerPoint (2011) to improve clarity and conciseness. Full size blots are uploaded in Additional File 8: Fig. S5. [file 12885_2020_6650_MOESM5_ESM.jpg]

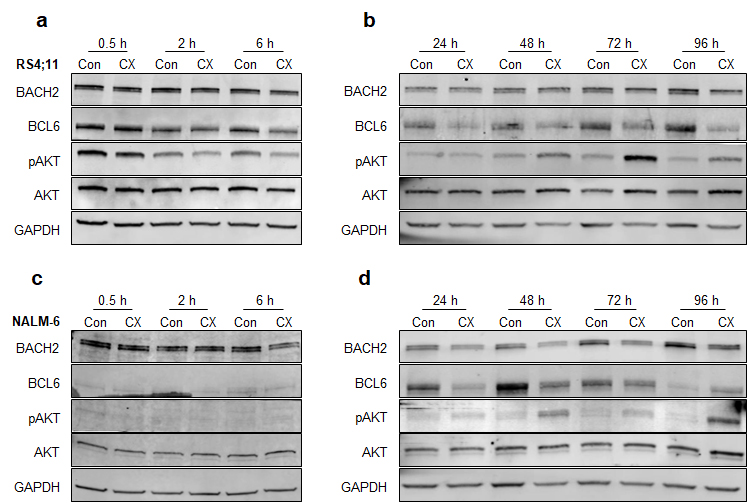

Supplement: Supplementary file 6 — Additional File 6: Figure S3. Evaluation of AKT phosphorylation, BACH2 and BCL6 protein expression in RS4;11 and NALM-6 cells. RS4;11 (a, b) and NALM-6 cells (c, d) were cultured and incubated with 5 μM CX-4945 or DMSO (control) for up to 96 h. Analysis of BACH2 and BCL6 protein expression as well as AKT phosphorylation was carried out by western blot with GAPDH as housekeeping gene. Representative images of three independent biological experiments. Short term effects of CX-4945 incubation were determined after 0.5 h, 2 h and 6 h (a, c). Long term effects of CX-4945 incubation were determined after 24Blots were processed and cropped using Image Studio Lite 5.2 software and MS PowerPoint (2011) to improve clarity and conciseness. Full size blots are uploaded in Additional File 8: Fig. S5. [file 12885_2020_6650_MOESM6_ESM.jpg]

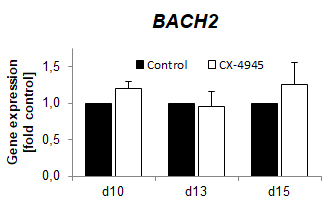

Supplement: Supplementary file 7 — Additional File 7: Figure S4. Gene expression analysis of BACH2. Bone marrow-derived leukemic blast populations of controls and treated mice sacrificed at d10, d13 and d15 were analyzed for changes in gene expression of BACH2 using qPCR. Mean values of ΔCT values from controls were calculated and set to 1 for each time point. ΔΔCT values were calculated for CX-4945-treated samples and compared to the respective time-matched control. Analyses were carried out in three technical replicates. Four animals per time point and study group; mean ± standard deviation. [file 12885_2020_6650_MOESM7_ESM.jpg]
