# Supplementary material for: Influence of Casein kinase II inhibitor CX-4945 on BCL6-mediated apoptotic signaling in B-ALL in vitro and in vivo
Source: BMC Cancer. 2020 Mar 4;20:184. doi: 10.1186/s12885-020-6650-9 (PMC7057698; doi:10.1186/s12885-020-6650-9)
Supplement: Supplementary file 3 — Additional File 3: Table S2. Gene expression fold changes of genes involved in apoptotic processes. [file 12885_2020_6650_MOESM3_ESM.docx]

Table S2: Gene expression fold changes of genes involved in apoptotic processes.

| Gene | Fold change d10 | Fold change d13 | Fold change d15 |
| --- | --- | --- | --- |
| BAD | -1,28 | -1,04 | -1,09 |
| BCL2 | 1,67 | -1,12 | 1,17 |
| BCL2L1 | -1,2 | 1,05 | -1,07 |
| BCL2L11 | 1,76 | 1,04 | 1,4 |
| BCL6 | -1,17 | -1,15 | -1,11 |
| BID | 1,02 | -1,02 | -1,17 |
| CASP9 | 1,46 | -1,2 | 1,03 |
| CHUK | -1,01 | 1,02 | -1,1 |
| IKBKB | -1,23 | -1,07 | -1,18 |
| IKBKG | 1,1 | -1,07 | -1,01 |
| MDM2 | 1,33 | -1,27 | -1,11 |
| NFKB1 | -1,52 | 1,34 | -1,13 |
| NFKBIA | 1,03 | -1,08 | -1,13 |
| RELA | -1,56 | -1,01 | -1,24 |
| TP53 | -1,28 | 1 | 1,13 |
