# Supplementary material for: Influence of Casein kinase II inhibitor CX-4945 on BCL6-mediated apoptotic signaling in B-ALL in vitro and in vivo
Source: BMC Cancer. 2020 Mar 4;20:184. doi: 10.1186/s12885-020-6650-9 (PMC7057698; doi:10.1186/s12885-020-6650-9)
Supplement: Supplementary file 4 — Additional File 4: Table S3. Gene expression fold changes and p-values of all genes included in the BCR/PI3K/CK2 panel. [file 12885_2020_6650_MOESM4_ESM.docx]

Table S3: Gene expression fold changes and p-values of all genes included in the BCR/PI3K/CK2 panel.

|  | d10 | | | | d13 | | | | d15 | | | | All time points | | | |
| --- | --- | --- | --- | --- | --- | --- | --- | --- | --- | --- | --- | --- | --- | --- | --- | --- |
| Gene | CX-4945 Avg (log2) | Control Avg (log2) | Fold Change | P-value | CX-4945 Avg (log2) | Control Avg (log2) | Fold Change | P-value | CX-4945 Avg (log2) | Control Avg (log2) | Fold Change | P-value | CX-4945 Avg (log2) | Control Avg (log2) | Fold Change | P-value |
| AKT1 | 11.61 | 12.01 | -1.32 | 0.1578 | 12.3 | 12.2 | 1.07 | 0.7095 | 12.09 | 12.28 | -1.14 | 0.4758 | 12 | 12.2 | -1.14 | 0.3739 |
| AKT1S1 | 7.6 | 8.33 | -1.66 | 0.283 | 8.6 | 8.86 | -1.19 | 0.6931 | 8.49 | 8.74 | -1.19 | 0.7031 | 8.49 | 8.78 | -1.22 | 0.2761 |
| AKT2 | 13.04 | 12.94 | 1.08 | 0.5552 | 13.08 | 13.06 | 1.01 | 0.9174 | 12.96 | 13.04 | -1.06 | 0.6524 | 13.02 | 13.01 | 1.01 | 0.8625 |
| AKT3 | 14.73 | 14.56 | 1.12 | 0.5663 | 13.91 | 13.75 | 1.12 | 0.5913 | 13.8 | 13.61 | 1.14 | 0.5127 | 14.09 | 13.94 | 1.1 | 0.5652 |
| ARAF | 11.42 | 11.36 | 1.04 | 0.6621 | 11.76 | 11.84 | -1.06 | 0.5504 | 11.86 | 11.83 | 1.02 | 0.8627 | 11.75 | 11.82 | -1.05 | 0.9982 |
| ATF2 | 12.78 | 12.31 | 1.39 | 0.0289 | 12.44 | 12.52 | -1.06 | 0.6431 | 12.65 | 12.35 | 1.23 | 0.124 | 12.61 | 12.41 | 1.14 | 0.0542 |
| BAD | 10.64 | 10.99 | -1.28 | 0.1078 | 11.28 | 11.34 | -1.04 | 0.7626 | 11.32 | 11.44 | -1.09 | 0.5397 | 11.24 | 11.35 | -1.08 | 0.3535 |
| BCL10 | 11.65 | 11.21 | 1.36 | 0.1915 | 11.43 | 11.83 | -1.32 | 0.236 | 11.63 | 11.44 | 1.14 | 0.5545 | 11.54 | 11.6 | -1.05 | 0.6761 |
| BCL2 | 13.07 | 12.33 | 1.67 | 0.0329 | 12.69 | 12.85 | -1.12 | 0.5572 | 12.77 | 12.55 | 1.17 | 0.4309 | 12.84 | 12.58 | 1.2 | 0.1487 |
| BCL2L1 | 9.83 | 10.1 | -1.2 | 0.2935 | 10.53 | 10.46 | 1.05 | 0.764 | 10.63 | 10.72 | -1.07 | 0.6924 | 10.58 | 10.54 | 1.03 | 0.6769 |
| BCL2L11 | 13.37 | 12.55 | 1.76 | 0.0288 | 12.8 | 12.75 | 1.04 | 0.8639 | 13.09 | 12.6 | 1.4 | 0.1387 | 12.96 | 12.66 | 1.23 | 0.023 |
| BCL6 | 7.51 | 7.73 | -1.17 | 0.6082 | 6.93 | 7.13 | -1.15 | 0.639 | 7.45 | 7.6 | -1.11 | 0.7245 | 7.31 | 7.5 | -1.14 | 0.4537 |
| BID | 10.92 | 10.89 | 1.02 | 0.7838 | 11.36 | 11.38 | -1.02 | 0.8385 | 11.42 | 11.65 | -1.17 | 0.08 | 11.26 | 11.33 | -1.05 | 0.687 |
| BLK | 13.9 | 13.69 | 1.16 | 0.1946 | 14 | 14.35 | -1.27 | 0.0528 | 14.24 | 14.24 | 1 | 0.9961 | 14.02 | 14.23 | -1.16 | 0.78 |
| BLNK | 5.04 | 5.74 | -1.62 | 0.1627 | 6.45 | 6.97 | -1.43 | 0.2842 | 6.7 | 6.98 | -1.21 | 0.5562 | 6.15 | 6.64 | -1.4 | 0.3097 |
| BRAF | 11.15 | 10.61 | 1.45 | 0.087 | 10.88 | 11.12 | -1.18 | 0.4039 | 11.25 | 11.02 | 1.17 | 0.4184 | 11.1 | 11.04 | 1.04 | 0.3184 |
| BTK | 12.45 | 12.65 | -1.15 | 0.397 | 13.11 | 13.04 | 1.05 | 0.7705 | 13.08 | 13.14 | -1.05 | 0.77 | 12.96 | 13.03 | -1.05 | 0.7369 |
| CALM1 | 13.4 | 13.56 | -1.12 | 0.4275 | 13.8 | 13.75 | 1.03 | 0.81 | 13.48 | 13.76 | -1.21 | 0.202 | 13.56 | 13.71 | -1.11 | 0.3134 |
| CALM2 | 15.07 | 14.33 | 1.67 | 0.154 | 14.04 | 13.74 | 1.23 | 0.5294 | 13.64 | 13.61 | 1.02 | 0.9512 | 14.22 | 13.74 | 1.39 | 0.3511 |
| CALM3 | 14.94 | 14.86 | 1.05 | 0.5251 | 15.03 | 15.26 | -1.17 | 0.0931 | 15.2 | 15.19 | 1.01 | 0.9064 | 15.05 | 15.19 | -1.11 | 0.6762 |
| CAMK2A | 0.69 | 0 | 1.61 | 0.3248 | 0.64 | 0 | 1.56 | 0.3556 | 0 | 0.59 | -1.51 | 0.3924 | 0 | 0 | -1 | 0.489 |
| CARD11 | 4.63 | 5.76 | -2.19 | 0.0995 | 6.2 | 6.19 | 1.01 | 0.9883 | 5.39 | 6.4 | -2.01 | 0.1341 | 5.53 | 6.33 | -1.74 | 0.1094 |
| CASP9 | 10.66 | 10.12 | 1.46 | 0.1301 | 10.24 | 10.5 | -1.2 | 0.432 | 10.45 | 10.41 | 1.03 | 0.8901 | 10.4 | 10.48 | -1.06 | 0.5465 |
| CASZ1 | 0 | 0 | -1 | 1 | 0 | 0 | -1 | 1 | 0 | 0 | -1 | 1 | 0 | 0 | -1 | 1 |
| CBL | 12.19 | 11.74 | 1.37 | 0.1151 | 11.93 | 12.11 | -1.13 | 0.4987 | 12.2 | 11.9 | 1.24 | 0.2561 | 12.09 | 11.99 | 1.07 | 0.1964 |
| CCND1 | 0 | 1.67 | -3.18 | 0.3438 | 2.89 | 2.56 | 1.26 | 0.8446 | 2.03 | 2.65 | -1.54 | 0.7158 | 1.54 | 2.58 | -2.06 | 0.4979 |
| CD19 | 12.38 | 10.94 | 2.71 | 0.0331 | 11.3 | 11.36 | -1.05 | 0.901 | 11.42 | 11.34 | 1.06 | 0.883 | 11.67 | 11.38 | 1.23 | 0.1738 |
| CD22 | 13.11 | 13.22 | -1.07 | 0.6843 | 13.26 | 13.39 | -1.09 | 0.6237 | 13.37 | 13.49 | -1.09 | 0.6153 | 13.32 | 13.45 | -1.09 | 0.3774 |
| CD79A | 12.62 | 13.29 | -1.59 | 0.0409 | 13.41 | 13.52 | -1.08 | 0.6838 | 13.59 | 13.64 | -1.04 | 0.85 | 13.42 | 13.51 | -1.06 | 0.259 |
| CD79B | 11.18 | 11.93 | -1.68 | 0.0088 | 12.16 | 12.21 | -1.04 | 0.7967 | 12.37 | 12.73 | -1.28 | 0.1153 | 12.21 | 12.27 | -1.04 | 0.2111 |
| CD81 | 13.05 | 13.79 | -1.68 | 0.0237 | 14.17 | 14.09 | 1.06 | 0.7573 | 14.23 | 14.41 | -1.14 | 0.4881 | 14.13 | 14.09 | 1.03 | 0.3536 |
| CDC42 | 15.76 | 14.61 | 2.22 | 0.0255 | 15.02 | 15.17 | -1.11 | 0.7173 | 15.13 | 15.07 | 1.04 | 0.8887 | 15.12 | 14.95 | 1.13 | 0.1914 |
| CDH1 | 1.3 | 1.75 | -1.37 | 0.6741 | 0.64 | 0 | 1.56 | 0.5524 | 0.68 | 0.71 | -1.02 | 0.9765 | 0.69 | 0.78 | -1.06 | 0.9279 |
| CDKN1A | 12.69 | 13.01 | -1.26 | 0.1328 | 12.93 | 13.18 | -1.19 | 0.2253 | 13.31 | 13.34 | -1.02 | 0.8748 | 12.96 | 13.26 | -1.22 | 0.2164 |
| CDKN1B | 12.04 | 12.93 | -1.86 | 0.0982 | 13.36 | 13.23 | 1.09 | 0.7945 | 13.15 | 13.34 | -1.14 | 0.6929 | 13.16 | 13.2 | -1.03 | 0.3657 |
| CHUK | 11.43 | 11.44 | -1.01 | 0.8501 | 11.47 | 11.44 | 1.02 | 0.6259 | 11.39 | 11.53 | -1.1 | 0.0316 | 11.43 | 11.45 | -1.01 | 0.2382 |
| CR2 | 1.48 | 1.11 | 1.29 | 0.8224 | 2.15 | 0.65 | 2.81 | 0.3843 | 1.29 | 1.3 | -1.01 | 0.9948 | 1.91 | 0.77 | 2.2 | 0.423 |
| CREB1 | 12.66 | 12.5 | 1.12 | 0.1664 | 12.59 | 12.62 | -1.02 | 0.7829 | 12.74 | 12.37 | 1.29 | 0.013 | 12.68 | 12.51 | 1.12 | 0.0391 |
| CRK | 14.66 | 14.09 | 1.49 | 0.303 | 12.72 | 12.35 | 1.29 | 0.4991 | 12.41 | 12.06 | 1.27 | 0.5206 | 12.81 | 12.7 | 1.08 | 0.517 |
| CRKL | 12.22 | 12.24 | -1.01 | 0.942 | 12.45 | 12.61 | -1.11 | 0.571 | 12.63 | 12.5 | 1.1 | 0.6141 | 12.44 | 12.58 | -1.1 | 0.9383 |
| CSNK1A1 | 13.65 | 13.3 | 1.27 | 0.2177 | 13.69 | 13.65 | 1.03 | 0.8846 | 13.53 | 13.69 | -1.11 | 0.5628 | 13.62 | 13.63 | -1 | 0.5949 |
| CSNK2A2 | 12.15 | 12 | 1.11 | 0.4771 | 12.3 | 12.39 | -1.07 | 0.6646 | 12.45 | 12.15 | 1.23 | 0.1931 | 12.29 | 12.18 | 1.07 | 0.373 |
| CSNK2B | 13.52 | 13.63 | -1.08 | 0.6554 | 13.9 | 13.99 | -1.06 | 0.748 | 13.89 | 14.06 | -1.13 | 0.4972 | 13.84 | 13.99 | -1.11 | 0.4594 |
| DAPP1 | 12.07 | 12.97 | -1.87 | 0.356 | 11.6 | 10.62 | 1.97 | 0.3218 | 10.6 | 10.61 | -1.01 | 0.9929 | 11.18 | 11.09 | 1.07 | 0.9762 |
| DEPTOR | 5.95 | 4.84 | 2.16 | 0.1464 | 5 | 4.88 | 1.09 | 0.8637 | 5.51 | 5.66 | -1.11 | 0.8298 | 5.41 | 5.13 | 1.22 | 0.3704 |
| E2F3 | 13.67 | 13.82 | -1.1 | 0.7133 | 12.77 | 12.45 | 1.25 | 0.4219 | 12.5 | 12.28 | 1.17 | 0.5647 | 12.95 | 12.43 | 1.44 | 0.7533 |
| EGF | 3.85 | 2.91 | 1.93 | 0.1905 | 4.54 | 4.58 | -1.02 | 0.9588 | 3.78 | 3.29 | 1.4 | 0.4771 | 4.2 | 3.48 | 1.64 | 0.332 |
| EGFR | 0.72 | 0 | 1.64 | 0.134 | 0 | 0 | -1 | 1 | 0 | 0 | -1 | 1 | 0 | 0 | -1 | 0.3409 |
| EIF4EBP1 | 13.04 | 13.03 | 1.01 | 0.9793 | 13.35 | 13.38 | -1.02 | 0.9102 | 13.37 | 13.55 | -1.13 | 0.5428 | 13.24 | 13.44 | -1.15 | 0.6992 |
| ELK1 | 9.78 | 9.21 | 1.49 | 0.0629 | 9.76 | 9.78 | -1.01 | 0.9362 | 9.93 | 10.05 | -1.08 | 0.6657 | 9.87 | 9.74 | 1.1 | 0.4733 |
| ERBB2 | 9.31 | 10.5 | -2.27 | 0.0695 | 10.15 | 10.41 | -1.19 | 0.6498 | 10.14 | 10.67 | -1.44 | 0.3618 | 9.87 | 10.53 | -1.58 | 0.0543 |
| ERBB3 | 3.07 | 3.72 | -1.58 | 0.6808 | 2.16 | 2.41 | -1.19 | 0.8735 | 3.18 | 0.91 | 4.79 | 0.1874 | 3.29 | 2.4 | 1.85 | 0.618 |
| ETS1 | 12.7 | 12.37 | 1.26 | 0.1861 | 12.66 | 12.58 | 1.06 | 0.7379 | 12.92 | 12.7 | 1.17 | 0.3531 | 12.78 | 12.57 | 1.15 | 0.1308 |
| FOXO1 | 8.75 | 8.77 | -1.01 | 0.9147 | 8.71 | 8.69 | 1.01 | 0.9321 | 9.27 | 8.91 | 1.28 | 0.1023 | 8.74 | 8.72 | 1.01 | 0.4389 |
| FOXO3 | 10.48 | 10.5 | -1.01 | 0.9455 | 10.98 | 10.9 | 1.05 | 0.7033 | 11.07 | 10.72 | 1.27 | 0.108 | 10.93 | 10.8 | 1.1 | 0.4131 |
| FOXO4 | 10.6 | 12.12 | -2.86 | 0.089 | 11.01 | 10.41 | 1.51 | 0.4577 | 10.49 | 10.55 | -1.04 | 0.9413 | 10.77 | 10.87 | -1.08 | 0.5213 |
| FYN | 10.94 | 11.11 | -1.13 | 0.4186 | 11.18 | 11.34 | -1.11 | 0.4621 | 11.19 | 11.12 | 1.06 | 0.7087 | 11.12 | 11.31 | -1.14 | 0.4811 |
| GAB1 | 11.73 | 10.99 | 1.67 | 0.1072 | 11.34 | 11.44 | -1.07 | 0.803 | 11.81 | 11.53 | 1.21 | 0.502 | 11.61 | 11.34 | 1.21 | 0.2011 |
| GAB2 | 12.27 | 12.46 | -1.14 | 0.2873 | 12.76 | 12.73 | 1.02 | 0.8401 | 12.98 | 12.87 | 1.08 | 0.5211 | 12.73 | 12.73 | 1 | 0.932 |
| GRB2 | 13.79 | 13.91 | -1.08 | 0.5475 | 14.36 | 14.26 | 1.07 | 0.6053 | 14.38 | 14.27 | 1.08 | 0.5543 | 14.21 | 14.24 | -1.02 | 0.8478 |
| GSK3A | 12.7 | 12.36 | 1.26 | 0.3035 | 12.53 | 12.61 | -1.06 | 0.7979 | 12.92 | 12.71 | 1.16 | 0.4973 | 12.72 | 12.62 | 1.07 | 0.3657 |
| GSK3B | 12.98 | 13.2 | -1.16 | 0.7086 | 12.08 | 11.41 | 1.6 | 0.2729 | 11.33 | 11.48 | -1.11 | 0.7988 | 12.05 | 11.63 | 1.33 | 0.8531 |
| GTF2I | 12.99 | 13.55 | -1.48 | 0.1567 | 13.85 | 13.91 | -1.04 | 0.8621 | 13.69 | 13.66 | 1.02 | 0.9365 | 13.68 | 13.74 | -1.04 | 0.4242 |
| HCLS1 | 13.31 | 13.4 | -1.07 | 0.4261 | 13.78 | 13.78 | 1 | 0.9553 | 13.68 | 13.71 | -1.02 | 0.8272 | 13.62 | 13.65 | -1.02 | 0.7762 |
| HIF1A | 12.82 | 13.66 | -1.78 | 0.0684 | 13.01 | 12.46 | 1.47 | 0.1895 | 12.36 | 12.54 | -1.13 | 0.6513 | 12.73 | 12.64 | 1.06 | 0.646 |
| HRAS | 8.31 | 8.67 | -1.28 | 0.4966 | 9.01 | 8.82 | 1.14 | 0.7141 | 8.54 | 9.03 | -1.41 | 0.3633 | 9 | 8.84 | 1.11 | 0.4296 |
| IKBKB | 11.47 | 11.77 | -1.23 | 0.3484 | 12.19 | 12.28 | -1.07 | 0.7559 | 12 | 12.25 | -1.18 | 0.4421 | 12.04 | 12.22 | -1.13 | 0.3455 |
| IKBKG | 10.98 | 10.84 | 1.1 | 0.6435 | 11.32 | 11.41 | -1.07 | 0.7626 | 11.32 | 11.33 | -1.01 | 0.9665 | 11.22 | 11.33 | -1.08 | 0.9491 |
| IL2RA | 0 | 0 | -1 | 1 | 0 | 0 | -1 | 1 | 0 | 0 | -1 | 1 | 0 | 0 | -1 | 1 |
| IL2RG | 12.28 | 12.07 | 1.16 | 0.2748 | 12.38 | 12.68 | -1.23 | 0.1365 | 12.61 | 12.94 | -1.25 | 0.1093 | 12.43 | 12.64 | -1.16 | 0.4834 |
| IL7 | 5.72 | 6.06 | -1.26 | 0.5153 | 5.82 | 6.28 | -1.38 | 0.3824 | 5.99 | 5.84 | 1.11 | 0.7661 | 5.92 | 6.08 | -1.12 | 0.3874 |
| IL7R | 5.83 | 6.54 | -1.64 | 0.0274 | 6.68 | 6.67 | 1.01 | 0.9596 | 7.17 | 6.65 | 1.43 | 0.0788 | 6.61 | 6.62 | -1 | 0.8295 |
| ILF2 | 13.31 | 13.93 | -1.54 | 0.0924 | 14.25 | 14.26 | -1.01 | 0.9691 | 14.19 | 14.42 | -1.17 | 0.4967 | 14.14 | 14.28 | -1.1 | 0.2851 |
| INPP5D | 11.36 | 11.13 | 1.17 | 0.3564 | 11.57 | 11.73 | -1.12 | 0.4998 | 12.02 | 11.7 | 1.24 | 0.2239 | 11.58 | 11.67 | -1.06 | 0.5448 |
| IRF4 | 9.02 | 8.72 | 1.23 | 0.5125 | 9.22 | 9.41 | -1.15 | 0.6624 | 9.86 | 9.56 | 1.23 | 0.5166 | 9.31 | 9.42 | -1.08 | 0.666 |
| IRS1 | 9.91 | 10.14 | -1.17 | 0.5019 | 9.8 | 10.1 | -1.23 | 0.3695 | 10.35 | 10.06 | 1.22 | 0.3884 | 9.97 | 10.06 | -1.07 | 0.6646 |
| JAK1 | 13.74 | 12.55 | 2.29 | 0.0421 | 12.75 | 12.91 | -1.12 | 0.7297 | 12.96 | 12.8 | 1.12 | 0.7353 | 12.88 | 12.83 | 1.03 | 0.2017 |
| JAK2 | 11.22 | 11.08 | 1.1 | 0.87 | 10.12 | 9.56 | 1.47 | 0.532 | 9.23 | 9.39 | -1.11 | 0.8589 | 9.92 | 9.6 | 1.25 | 0.7769 |
| JAK3 | 11.13 | 11.61 | -1.39 | 0.1226 | 11.69 | 11.81 | -1.09 | 0.6588 | 11.71 | 11.84 | -1.09 | 0.6583 | 11.66 | 11.8 | -1.1 | 0.197 |
| JUN | 3.55 | 3.87 | -1.25 | 0.7463 | 4.62 | 4.41 | 1.15 | 0.8342 | 4.64 | 4.72 | -1.06 | 0.9352 | 3.8 | 4.78 | -1.97 | 0.9007 |
| KLF11 | 1.27 | 0 | 2.41 | 0.1814 | 0 | 0 | -1 | 1 | 0 | 0.71 | -1.64 | 0.4303 | 0 | 0 | -1 | 0.7085 |
| KRAS | 14.04 | 14.58 | -1.45 | 0.2342 | 13.78 | 13.03 | 1.69 | 0.1134 | 13.04 | 12.94 | 1.07 | 0.8099 | 13.59 | 13.08 | 1.43 | 0.8092 |
| LAT2 | 10.46 | 10.85 | -1.31 | 0.2671 | 11.34 | 11.4 | -1.05 | 0.8451 | 11.16 | 11.39 | -1.18 | 0.4805 | 11.03 | 11.29 | -1.19 | 0.3766 |
| LCK | 8.82 | 9.4 | -1.49 | 0.1078 | 9.53 | 9.64 | -1.08 | 0.731 | 9.76 | 10.14 | -1.3 | 0.2528 | 9.48 | 9.68 | -1.15 | 0.2076 |
| LYN | 12.29 | 12.12 | 1.12 | 0.3866 | 12.34 | 12.32 | 1.01 | 0.9065 | 12.29 | 12.45 | -1.12 | 0.3848 | 12.32 | 12.38 | -1.04 | 0.9463 |
| MALT1 | 10.52 | 10.13 | 1.31 | 0.0393 | 10.51 | 10.64 | -1.09 | 0.4211 | 10.37 | 10.54 | -1.12 | 0.2985 | 10.48 | 10.55 | -1.05 | 0.8067 |
| MAP2K1 | 12.48 | 12.73 | -1.19 | 0.2657 | 12.51 | 12.4 | 1.08 | 0.6149 | 12.37 | 12.4 | -1.02 | 0.8807 | 12.44 | 12.49 | -1.04 | 0.6324 |
| MAP2K2 | 11.52 | 11.72 | -1.15 | 0.3397 | 11.97 | 11.96 | 1.01 | 0.9672 | 12.02 | 12.14 | -1.09 | 0.5465 | 11.88 | 12.01 | -1.1 | 0.5099 |
| MAP2K3 | 11.59 | 11.1 | 1.4 | 0.077 | 11.36 | 11.52 | -1.12 | 0.5 | 11.47 | 11.51 | -1.02 | 0.8823 | 11.44 | 11.46 | -1.02 | 0.511 |
| MAP2K4 | 13.54 | 14.05 | -1.42 | 0.3756 | 12.53 | 11.76 | 1.71 | 0.1909 | 11.77 | 12.03 | -1.19 | 0.646 | 12.59 | 11.96 | 1.55 | 0.9924 |
| MAP2K5 | 9.93 | 10.31 | -1.3 | 0.1814 | 10.54 | 10.78 | -1.18 | 0.3799 | 10.4 | 10.32 | 1.06 | 0.7646 | 10.41 | 10.52 | -1.08 | 0.3639 |
| MAP2K6 | 9.05 | 9.62 | -1.48 | 0.0819 | 10.11 | 9.98 | 1.09 | 0.6472 | 9.87 | 9.98 | -1.08 | 0.68 | 9.86 | 9.87 | -1.01 | 0.4761 |
| MAP2K7 | 12.39 | 11.71 | 1.6 | 0.0914 | 11.96 | 12.01 | -1.04 | 0.8865 | 12.03 | 12.04 | -1.01 | 0.9746 | 11.98 | 12 | -1.01 | 0.2934 |
| MAP3K7 | 12.45 | 11.99 | 1.37 | 0.0505 | 12.46 | 12.46 | 1 | 0.9811 | 12.44 | 12.38 | 1.05 | 0.7342 | 12.45 | 12.27 | 1.13 | 0.184 |
| MAP4K1 | 12.27 | 11.34 | 1.9 | 0.0513 | 11.64 | 11.83 | -1.15 | 0.6271 | 11.98 | 11.82 | 1.12 | 0.6839 | 11.79 | 11.72 | 1.05 | 0.2235 |
| MAPK1 | 14.47 | 14.18 | 1.22 | 0.26 | 13.94 | 13.87 | 1.05 | 0.751 | 13.99 | 13.91 | 1.06 | 0.7459 | 14.05 | 13.91 | 1.1 | 0.3793 |
| MAPK14 | 12.39 | 12.68 | -1.22 | 0.0169 | 12.87 | 12.92 | -1.04 | 0.5617 | 12.85 | 12.76 | 1.06 | 0.3455 | 12.86 | 12.79 | 1.05 | 0.4864 |
| MAPK3 | 12.22 | 11.32 | 1.87 | 0.0214 | 11.65 | 11.79 | -1.11 | 0.6289 | 12.04 | 11.94 | 1.07 | 0.76 | 11.85 | 11.74 | 1.08 | 0.2021 |
| MAPK4 | 0.72 | 0 | 1.64 | 0.5408 | 0.64 | 1.65 | -2.01 | 0.3967 | 0 | 0 | -1 | 1 | 0 | 0 | -1 | 0.8788 |
| MAPK8 | 11.56 | 11.07 | 1.4 | 0.1827 | 11.37 | 11.46 | -1.07 | 0.7846 | 11.66 | 11.42 | 1.19 | 0.471 | 11.54 | 11.41 | 1.09 | 0.2434 |
| MAPK9 | 11.27 | 10.9 | 1.3 | 0.31 | 11.09 | 11.56 | -1.39 | 0.2122 | 11.34 | 11.22 | 1.08 | 0.7438 | 11.25 | 11.43 | -1.13 | 0.9758 |
| MAPKAP1 | 12.7 | 13.97 | -2.41 | 0.0722 | 13.05 | 12.65 | 1.31 | 0.5237 | 12.41 | 12.7 | -1.23 | 0.633 | 12.72 | 12.95 | -1.17 | 0.3472 |
| MAX | 12.61 | 12.67 | -1.05 | 0.8014 | 12.66 | 12.69 | -1.02 | 0.912 | 12.77 | 12.77 | -1 | 0.9978 | 12.75 | 12.71 | 1.03 | 0.7928 |
| MDM2 | 12.66 | 12.25 | 1.33 | 0.1646 | 12.44 | 12.79 | -1.27 | 0.2332 | 12.53 | 12.69 | -1.11 | 0.5742 | 12.42 | 12.68 | -1.2 | 0.8602 |
| MEF2C | 14.34 | 15.08 | -1.67 | 0.0224 | 15.53 | 15.54 | -1.01 | 0.9589 | 15.73 | 15.57 | 1.12 | 0.5244 | 15.62 | 15.52 | 1.07 | 0.5442 |
| MEF2D | 13.54 | 12.84 | 1.63 | 0.1645 | 13.2 | 13.2 | -1 | 0.9976 | 13.46 | 13.3 | 1.12 | 0.7332 | 13.29 | 13.21 | 1.05 | 0.235 |
| MLST8 | 10.7 | 11 | -1.23 | 0.2975 | 11.13 | 11.35 | -1.17 | 0.4298 | 11.01 | 11.31 | -1.23 | 0.2969 | 10.99 | 11.27 | -1.22 | 0.1138 |
| MTOR | 11.92 | 12.21 | -1.22 | 0.2577 | 12.58 | 12.56 | 1.02 | 0.917 | 12.2 | 12.35 | -1.11 | 0.5359 | 12.27 | 12.41 | -1.1 | 0.4414 |
| NCK1 | 10.58 | 10.89 | -1.24 | 0.1525 | 10.85 | 10.78 | 1.05 | 0.7346 | 11.05 | 10.85 | 1.15 | 0.3402 | 10.87 | 10.81 | 1.04 | 0.9008 |
| NF1 | 11.47 | 11.6 | -1.1 | 0.6225 | 11.83 | 11.99 | -1.11 | 0.5792 | 11.93 | 11.62 | 1.24 | 0.293 | 11.84 | 11.79 | 1.04 | 0.9805 |
| NFATC2 | 9.6 | 9.14 | 1.38 | 0.1766 | 9.22 | 9.38 | -1.12 | 0.5996 | 9.57 | 9.35 | 1.17 | 0.4872 | 9.41 | 9.3 | 1.08 | 0.3147 |
| NFATC3 | 14.65 | 14.3 | 1.27 | 0.6176 | 13.45 | 13.1 | 1.28 | 0.617 | 13.14 | 13.02 | 1.09 | 0.8652 | 13.68 | 13.23 | 1.37 | 0.5906 |
| NFKB1 | 11.85 | 12.45 | -1.52 | 0.1913 | 11.9 | 11.48 | 1.34 | 0.3494 | 11.36 | 11.54 | -1.13 | 0.6753 | 11.62 | 11.6 | 1.01 | 0.6824 |
| NFKBIA | 11.17 | 11.13 | 1.03 | 0.869 | 11.52 | 11.64 | -1.08 | 0.6696 | 11.51 | 11.69 | -1.13 | 0.507 | 11.4 | 11.62 | -1.17 | 0.6446 |
| NRAS | 13.25 | 13.2 | 1.04 | 0.8466 | 13.4 | 13.46 | -1.04 | 0.8312 | 13.72 | 13.62 | 1.08 | 0.7071 | 13.5 | 13.42 | 1.06 | 0.8453 |
| PDK1 | 10.85 | 11.08 | -1.18 | 0.226 | 11.53 | 11.51 | 1.02 | 0.9008 | 11.17 | 11.35 | -1.13 | 0.3351 | 11.17 | 11.38 | -1.16 | 0.451 |
| PDPK1 | 10.63 | 10.63 | 1 | 0.9939 | 10.83 | 10.85 | -1.02 | 0.8968 | 10.87 | 10.92 | -1.04 | 0.7592 | 10.77 | 10.88 | -1.08 | 0.8156 |
| PIK3AP1 | 14.44 | 14.31 | 1.09 | 0.6191 | 14.57 | 14.64 | -1.06 | 0.7486 | 14.72 | 14.88 | -1.12 | 0.5154 | 14.73 | 14.63 | 1.07 | 0.8073 |
| PIK3CA | 13.74 | 14.28 | -1.46 | 0.2189 | 12.88 | 12.36 | 1.43 | 0.2434 | 12.28 | 12.07 | 1.16 | 0.6082 | 12.92 | 12.28 | 1.56 | 0.9126 |
| PIK3CB | 12.2 | 10.82 | 2.61 | 0.0702 | 11.11 | 11.48 | -1.29 | 0.5773 | 11.61 | 11.21 | 1.32 | 0.5469 | 11.23 | 11.17 | 1.04 | 0.2264 |
| PIK3CD | 11.05 | 11.7 | -1.56 | 0.1373 | 11.9 | 11.92 | -1.02 | 0.95 | 11.96 | 12 | -1.03 | 0.9271 | 11.86 | 11.89 | -1.02 | 0.3819 |
| PIK3CG | 12.53 | 11.42 | 2.16 | 0.0674 | 11.79 | 11.93 | -1.1 | 0.7965 | 11.98 | 11.75 | 1.17 | 0.6621 | 11.85 | 11.67 | 1.14 | 0.1809 |
| PIK3R1 | 13.35 | 12.4 | 1.93 | 0.0535 | 12.93 | 12.79 | 1.11 | 0.7233 | 13.03 | 12.55 | 1.39 | 0.2716 | 12.95 | 12.55 | 1.32 | 0.0295 |
| PIP5K1A | 11.73 | 11.48 | 1.19 | 0.5254 | 12.12 | 12.2 | -1.06 | 0.8393 | 12.16 | 12.19 | -1.02 | 0.9475 | 12.11 | 12.17 | -1.04 | 0.8435 |
| PIP5K1B | 7.91 | 8.14 | -1.17 | 0.0874 | 8.76 | 8.97 | -1.16 | 0.1032 | 9.4 | 8.94 | 1.37 | 0.0058 | 8.71 | 8.94 | -1.17 | 0.9812 |
| PIP5K1C | 13.08 | 14.4 | -2.49 | 0.1095 | 12.98 | 12.18 | 1.74 | 0.2997 | 11.8 | 11.93 | -1.1 | 0.8539 | 12.54 | 12.57 | -1.02 | 0.7404 |
| PLCG1 | 9.16 | 8.59 | 1.49 | 0.1833 | 9.04 | 8.88 | 1.12 | 0.6878 | 8.79 | 8.63 | 1.12 | 0.6779 | 8.93 | 8.77 | 1.12 | 0.1503 |
| PLCG2 | 12.05 | 12.41 | -1.28 | 0.1711 | 12.59 | 12.81 | -1.16 | 0.3949 | 12.51 | 12.78 | -1.2 | 0.2941 | 12.49 | 12.66 | -1.12 | 0.1302 |
| PRKCA | 9.93 | 9.26 | 1.6 | 0.0441 | 9.32 | 9.73 | -1.33 | 0.1745 | 9.75 | 9.25 | 1.41 | 0.1103 | 9.69 | 9.25 | 1.35 | 0.2173 |
| PRKCB | 10.8 | 10.92 | -1.09 | 0.4349 | 11.34 | 11.6 | -1.2 | 0.1167 | 11.19 | 11.38 | -1.14 | 0.2393 | 11.16 | 11.32 | -1.11 | 0.3077 |
| PRKCD | 11.03 | 11.68 | -1.57 | 0.2074 | 12.01 | 12.09 | -1.06 | 0.8694 | 11.87 | 12.08 | -1.16 | 0.6594 | 11.88 | 12.01 | -1.09 | 0.3111 |
| PRKCE | 11.54 | 12.14 | -1.52 | 0.0654 | 12.09 | 12.14 | -1.03 | 0.8601 | 12.15 | 12.04 | 1.08 | 0.7031 | 12.07 | 12.11 | -1.03 | 0.3176 |
| PRKCZ | 2.26 | 0 | 4.8 | 0.2418 | 1.21 | 1.9 | -1.62 | 0.7026 | 0.71 | 1.3 | -1.5 | 0.7476 | 0.64 | 0.76 | -1.09 | 0.7261 |
| PRR5 | 0 | 0 | -1 | 1 | 0 | 0 | -1 | 1 | 0 | 0 | -1 | 1 | 0 | 0 | -1 | 1 |
| PRR5L | 8.85 | 9.08 | -1.17 | 0.4791 | 8.88 | 9.33 | -1.37 | 0.1849 | 9.53 | 9.07 | 1.38 | 0.1762 | 9.08 | 9.18 | -1.07 | 0.7279 |
| PSEN2 | 0 | 0.75 | -1.68 | 0.3211 | 0.64 | 0 | 1.56 | 0.3875 | 0.68 | 0 | 1.6 | 0.3644 | 0 | 0 | -1 | 0.6196 |
| PTEN | 12.85 | 12.26 | 1.5 | 0.1446 | 12.54 | 12.73 | -1.14 | 0.603 | 12.68 | 12.55 | 1.09 | 0.7253 | 12.61 | 12.55 | 1.04 | 0.3739 |
| PTK2B | 14.3 | 15.14 | -1.78 | 0.0548 | 14.94 | 14.87 | 1.05 | 0.8456 | 14.75 | 14.83 | -1.06 | 0.8207 | 14.84 | 14.96 | -1.09 | 0.2015 |
| PTPN11 | 13.4 | 13.3 | 1.08 | 0.4651 | 13.49 | 13.64 | -1.1 | 0.3307 | 13.47 | 13.42 | 1.03 | 0.754 | 13.47 | 13.47 | 1 | 0.9803 |
| PTPN18 | 11.7 | 12 | -1.23 | 0.2095 | 12.27 | 12.4 | -1.09 | 0.5626 | 12.25 | 12.28 | -1.02 | 0.8966 | 12.13 | 12.33 | -1.14 | 0.3925 |
| PTPN6 | 13.51 | 13.25 | 1.2 | 0.19 | 13.64 | 13.84 | -1.15 | 0.2821 | 14.13 | 14 | 1.09 | 0.4949 | 13.76 | 13.86 | -1.07 | 0.7748 |
| PTPRC | 6.87 | 6.83 | 1.03 | 0.8741 | 7.47 | 7.33 | 1.11 | 0.6309 | 7.54 | 7.48 | 1.04 | 0.8522 | 7.34 | 7.26 | 1.06 | 0.7187 |
| RAC1 | 12.81 | 12.92 | -1.08 | 0.7033 | 13.34 | 13.12 | 1.16 | 0.4457 | 12.94 | 13.22 | -1.21 | 0.3457 | 13.06 | 13.18 | -1.08 | 0.7511 |
| RAC2 | 13.54 | 13.85 | -1.24 | 0.0381 | 14.09 | 14.26 | -1.12 | 0.1937 | 14.25 | 14.45 | -1.15 | 0.1336 | 14.07 | 14.23 | -1.12 | 0.2477 |
| RAF1 | 12.06 | 12.76 | -1.63 | 0.1541 | 13.18 | 13.21 | -1.02 | 0.9497 | 13 | 13.06 | -1.04 | 0.8911 | 13 | 13.05 | -1.04 | 0.4039 |
| RAPGEF1 | 11.91 | 12.36 | -1.36 | 0.1825 | 12.67 | 12.65 | 1.01 | 0.9494 | 12.48 | 12.5 | -1.01 | 0.9456 | 12.52 | 12.59 | -1.05 | 0.4787 |
| RASGRP3 | 4.02 | 2.52 | 2.82 | 0.3666 | 3.89 | 4.89 | -2 | 0.5393 | 4.77 | 3.66 | 2.16 | 0.4955 | 4.12 | 4.45 | -1.26 | 0.5318 |
| REL | 10.14 | 9.94 | 1.15 | 0.3091 | 10.17 | 10.27 | -1.07 | 0.6089 | 10.42 | 10.04 | 1.3 | 0.0833 | 10.26 | 10.06 | 1.15 | 0.1967 |
| RELA | 11.46 | 12.11 | -1.56 | 0.1933 | 12.53 | 12.54 | -1.01 | 0.9794 | 12.41 | 12.72 | -1.24 | 0.5121 | 12.38 | 12.56 | -1.13 | 0.3282 |
| RICTOR | 11.08 | 11.13 | -1.03 | 0.8347 | 11.66 | 11.7 | -1.03 | 0.8656 | 11.58 | 11.37 | 1.16 | 0.3471 | 11.6 | 11.43 | 1.13 | 0.8164 |
| RPS6KA1 | 12.78 | 12.55 | 1.18 | 0.0677 | 12.73 | 12.94 | -1.15 | 0.1002 | 12.81 | 12.95 | -1.1 | 0.258 | 12.78 | 12.9 | -1.09 | 0.7328 |
| RPS6KA3 | 12.66 | 12.42 | 1.19 | 0.5926 | 12.68 | 12.71 | -1.02 | 0.9503 | 12.68 | 12.83 | -1.12 | 0.7287 | 12.68 | 12.7 | -1.01 | 0.9267 |
| RPS6KB1 | 11.19 | 10.59 | 1.51 | 0.0464 | 11.05 | 10.94 | 1.08 | 0.6648 | 11.11 | 10.93 | 1.13 | 0.4946 | 11.11 | 10.94 | 1.12 | 0.0504 |
| RPTOR | 11.58 | 12.18 | -1.52 | 0.2608 | 11.57 | 11.57 | -1 | 0.9989 | 11.36 | 11.33 | 1.02 | 0.9535 | 11.46 | 11.56 | -1.07 | 0.5007 |
| SH3BP2 | 9.31 | 9.24 | 1.05 | 0.9057 | 9.75 | 9.72 | 1.02 | 0.9541 | 9.56 | 9.68 | -1.09 | 0.8303 | 9.78 | 9.7 | 1.05 | 0.9793 |
| SHC1 | 11.8 | 11.89 | -1.06 | 0.6057 | 12.23 | 12.24 | -1.01 | 0.9519 | 12.3 | 12.28 | 1.01 | 0.9188 | 12.13 | 12.23 | -1.07 | 0.8546 |
| SMAD2 | 12.05 | 11.57 | 1.4 | 0.0217 | 11.74 | 11.85 | -1.09 | 0.4772 | 11.52 | 11.44 | 1.06 | 0.6102 | 11.72 | 11.62 | 1.07 | 0.3206 |
| SMAD3 | 7.19 | 6.97 | 1.16 | 0.5843 | 6.9 | 6.27 | 1.55 | 0.1465 | 7.12 | 7.38 | -1.19 | 0.5273 | 7.07 | 6.9 | 1.12 | 0.4808 |
| SMAD4 | 11.9 | 11.57 | 1.26 | 0.1652 | 11.77 | 11.89 | -1.08 | 0.6126 | 11.89 | 11.82 | 1.05 | 0.7572 | 11.82 | 11.83 | -1 | 0.424 |
| SOS1 | 9.68 | 9.47 | 1.16 | 0.6888 | 10.01 | 9.74 | 1.2 | 0.6095 | 10.09 | 9.71 | 1.3 | 0.4799 | 10.11 | 9.65 | 1.38 | 0.269 |
| SPRY2 | 11.1 | 10.31 | 1.72 | 0.0315 | 10.95 | 10.95 | 1 | 0.9985 | 11.25 | 10.98 | 1.21 | 0.374 | 11.06 | 10.88 | 1.13 | 0.0999 |
| STAT1 | 12.59 | 12.25 | 1.26 | 0.1785 | 12.37 | 12.51 | -1.1 | 0.5441 | 12.48 | 12.46 | 1.02 | 0.9126 | 12.4 | 12.44 | -1.03 | 0.5475 |
| STAT2 | 10.03 | 10.62 | -1.5 | 0.111 | 10.69 | 10.83 | -1.1 | 0.6773 | 10.85 | 10.65 | 1.15 | 0.5488 | 10.72 | 10.67 | 1.03 | 0.438 |
| STAT3 | 12.99 | 13.68 | -1.62 | 0.0957 | 12.52 | 11.97 | 1.46 | 0.1728 | 11.77 | 11.99 | -1.16 | 0.5589 | 12.44 | 12.11 | 1.26 | 0.791 |
| STAT4 | 4.79 | 5.51 | -1.66 | 0.3234 | 4.99 | 6.08 | -2.13 | 0.1572 | 5.54 | 5.47 | 1.05 | 0.9138 | 5.12 | 5.63 | -1.43 | 0.1346 |
| STAT5A | 9.3 | 9.2 | 1.07 | 0.7427 | 9.19 | 9.15 | 1.03 | 0.8938 | 9.66 | 9.32 | 1.26 | 0.2946 | 9.41 | 9.17 | 1.18 | 0.3491 |
| STAT5B | 10.13 | 10.51 | -1.3 | 0.1143 | 10.9 | 10.86 | 1.03 | 0.8633 | 10.88 | 10.9 | -1.02 | 0.915 | 10.74 | 10.85 | -1.08 | 0.5564 |
| STAT6 | 12.06 | 12.29 | -1.17 | 0.3461 | 12.56 | 12.68 | -1.09 | 0.5947 | 12.61 | 12.8 | -1.14 | 0.441 | 12.54 | 12.62 | -1.06 | 0.3379 |
| SYK | 13.23 | 13.08 | 1.11 | 0.0992 | 13.52 | 13.51 | 1 | 0.9728 | 13.53 | 13.55 | -1.02 | 0.7269 | 13.5 | 13.53 | -1.02 | 0.7413 |
| TEC | 7.71 | 8.43 | -1.65 | 0.2331 | 8.63 | 8.83 | -1.15 | 0.7319 | 8.96 | 8.89 | 1.05 | 0.8992 | 8.65 | 8.82 | -1.12 | 0.4419 |
| TGFB2 | 4.22 | 3.8 | 1.34 | 0.8243 | 3.17 | 2.8 | 1.29 | 0.8454 | 2.07 | 4.1 | -4.07 | 0.3039 | 3.53 | 3.99 | -1.38 | 0.6659 |
| TP53 | 12.41 | 12.76 | -1.28 | 0.1496 | 13.08 | 13.07 | 1 | 0.9809 | 13.3 | 13.13 | 1.13 | 0.4504 | 13.04 | 13.08 | -1.03 | 0.7849 |
| TYK2 | 11.56 | 11.65 | -1.07 | 0.7135 | 11.98 | 12.15 | -1.13 | 0.52 | 11.94 | 11.96 | -1.02 | 0.9305 | 11.87 | 11.98 | -1.08 | 0.577 |
| USP7 | 13.38 | 13.36 | 1.01 | 0.9097 | 13.46 | 13.46 | 1 | 0.9993 | 13.36 | 13.29 | 1.04 | 0.719 | 13.4 | 13.33 | 1.05 | 0.7457 |
| VAV1 | 12.18 | 12.43 | -1.19 | 0.3123 | 12.84 | 12.86 | -1.02 | 0.9254 | 12.62 | 13.02 | -1.32 | 0.1308 | 12.62 | 12.87 | -1.19 | 0.2738 |
| VAV2 | 11.48 | 11.64 | -1.12 | 0.337 | 11.99 | 12.16 | -1.13 | 0.3058 | 12.12 | 11.97 | 1.11 | 0.36 | 12 | 11.99 | 1.01 | 0.7344 |
| VEGFA | 5.66 | 5.16 | 1.41 | 0.6932 | 6.27 | 6.26 | 1.01 | 0.9902 | 6.55 | 6.4 | 1.11 | 0.9054 | 6.12 | 6.41 | -1.22 | 0.7275 |
