# Supplementary material for: Influence of Casein kinase II inhibitor CX-4945 on BCL6-mediated apoptotic signaling in B-ALL in vitro and in vivo
Source: BMC Cancer. 2020 Mar 4;20:184. doi: 10.1186/s12885-020-6650-9 (PMC7057698; doi:10.1186/s12885-020-6650-9)

Original Blots Figure 3b

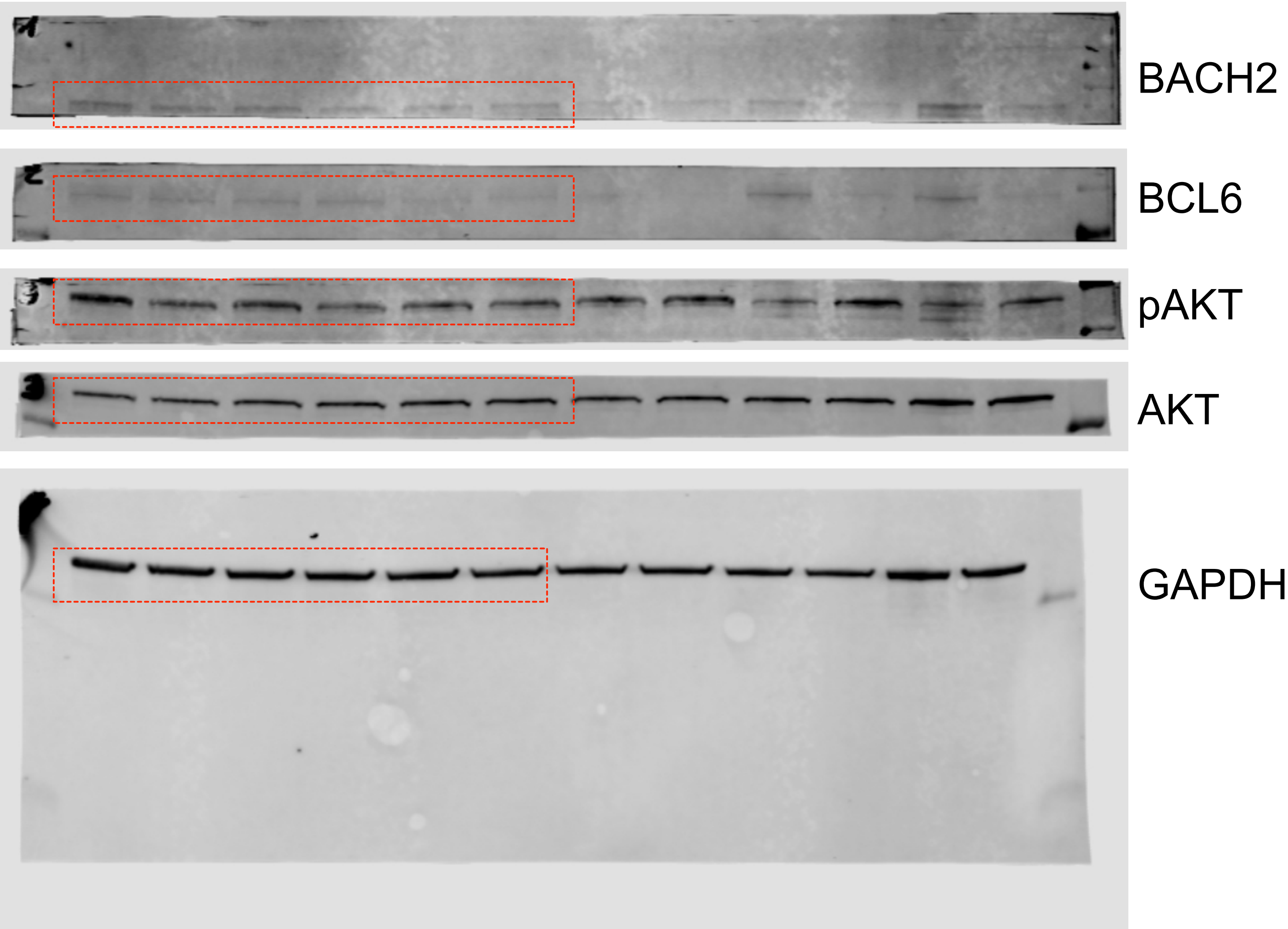

Original Blots Figure 3c

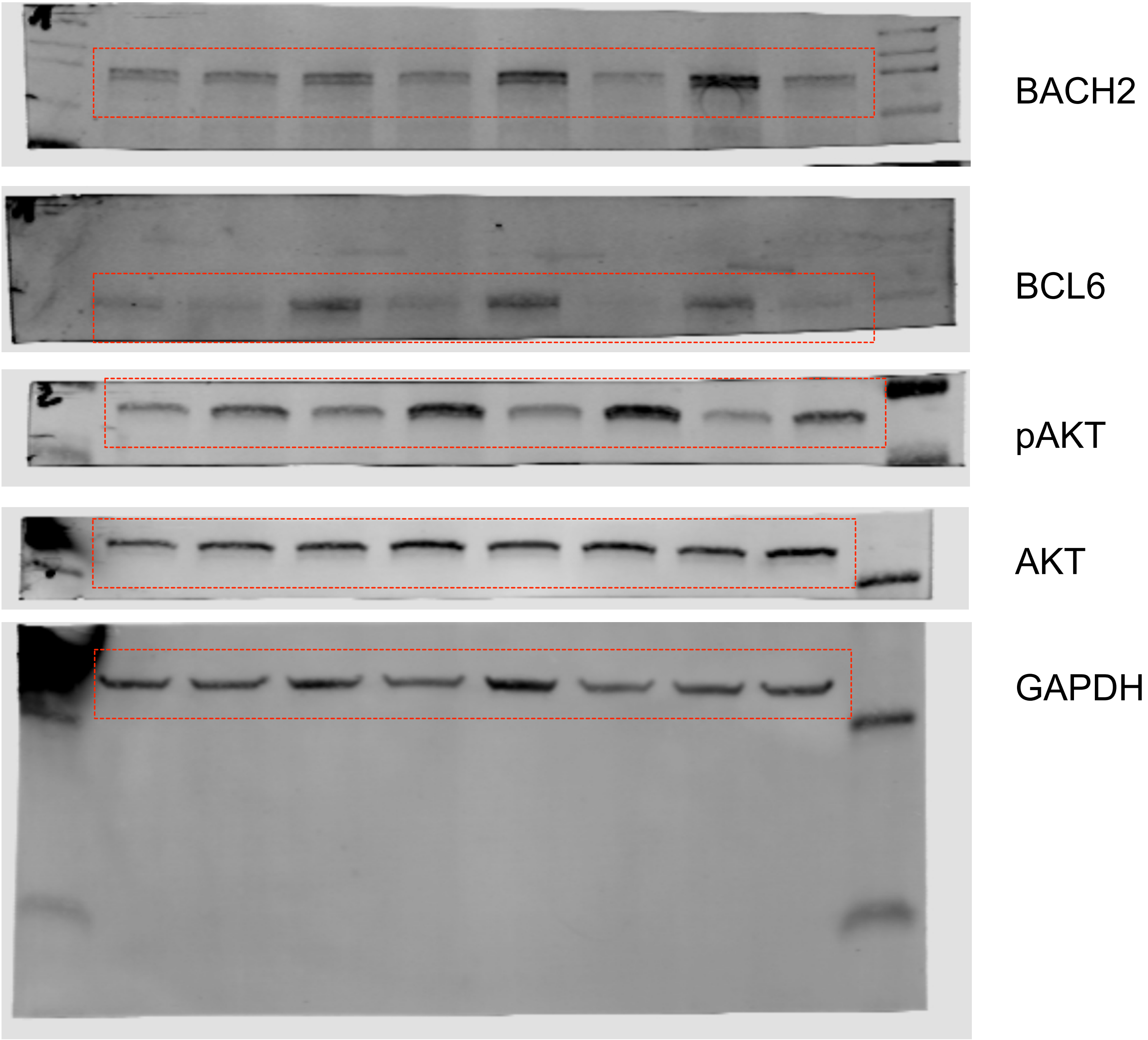

Original Blots Figure S2

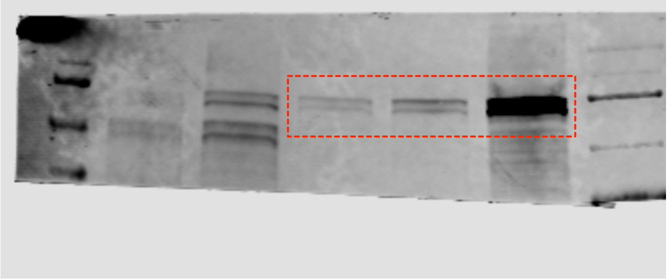

BACH2

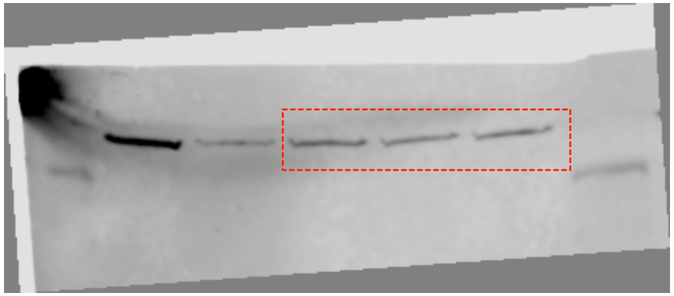

GAPDH

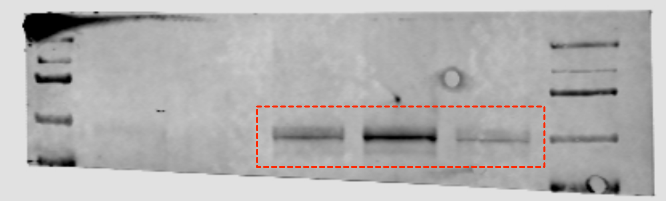

BCL6

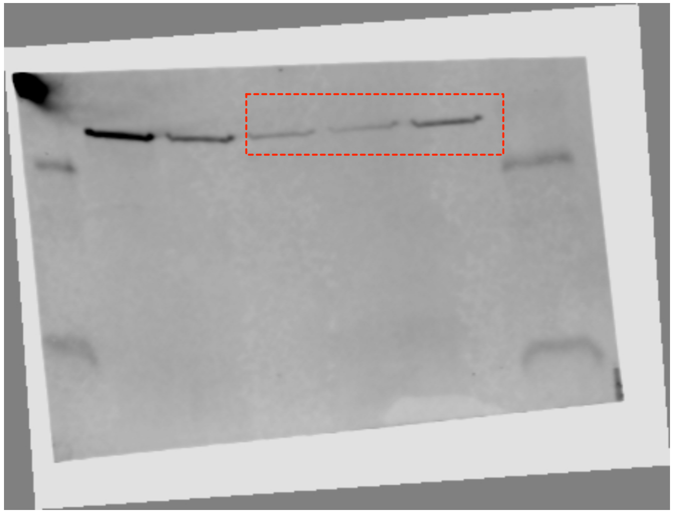

GAPDH

Original Blots Figure S3a

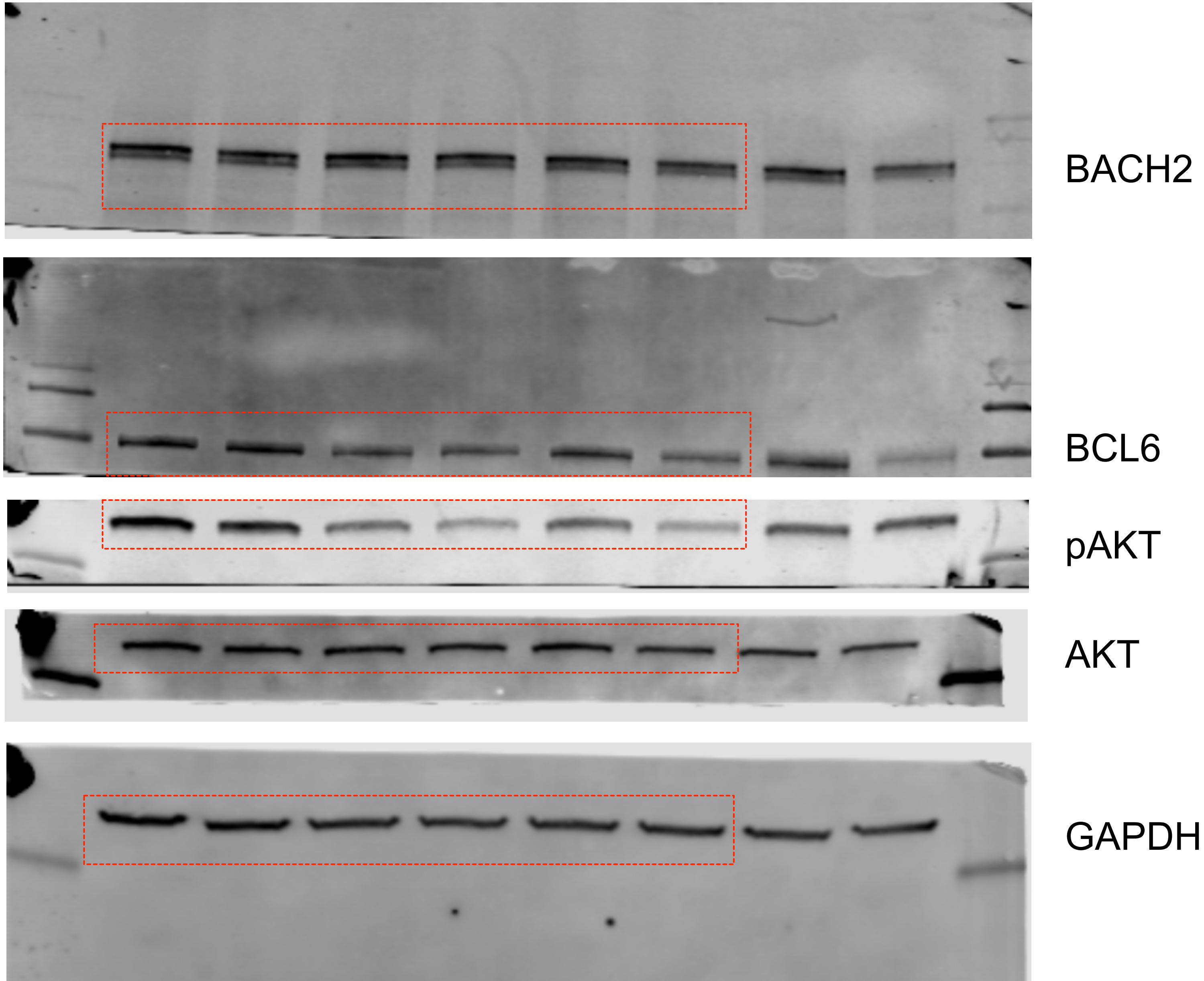

Original Blots Figure S3b

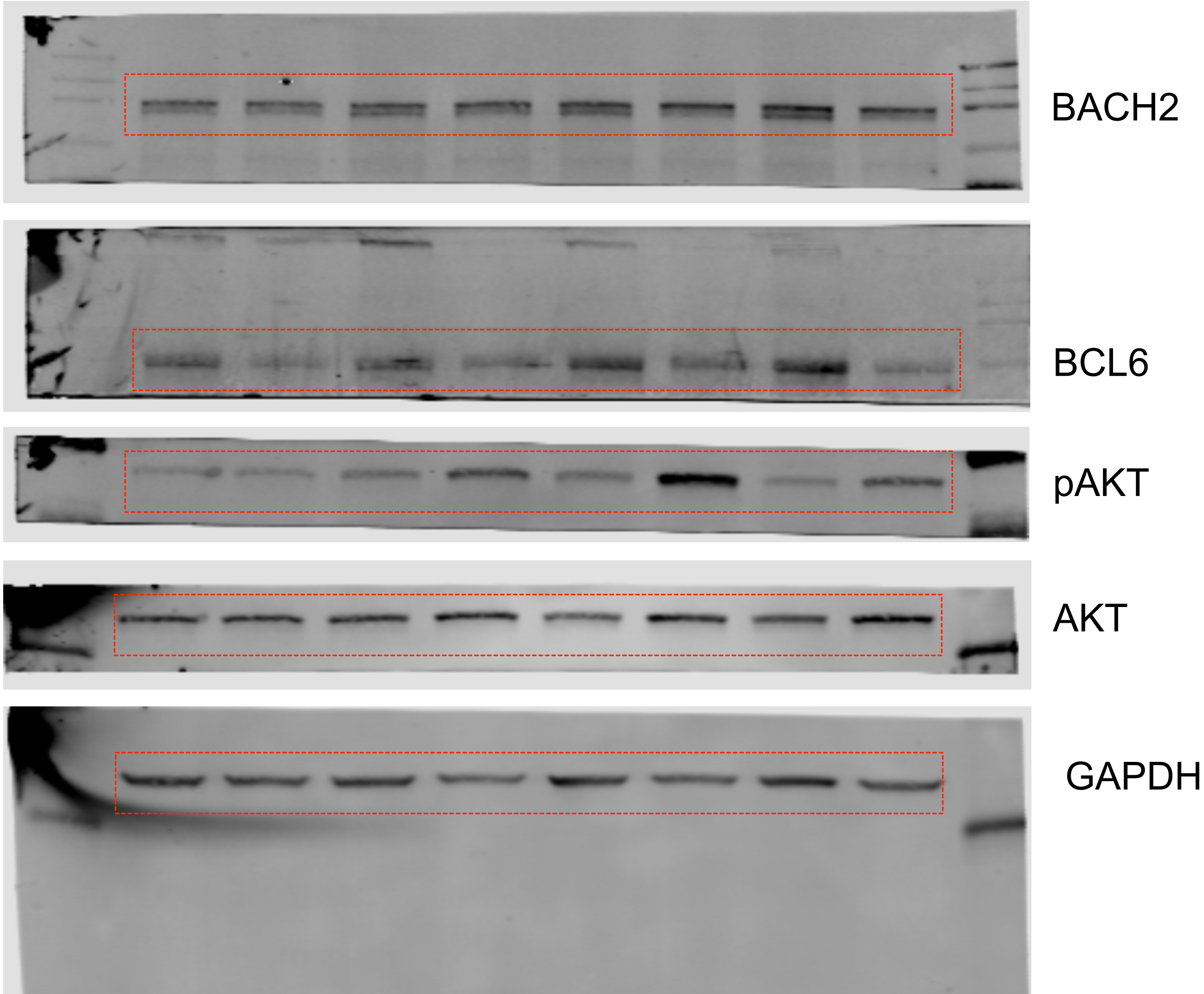

Original Blots Figure S3c

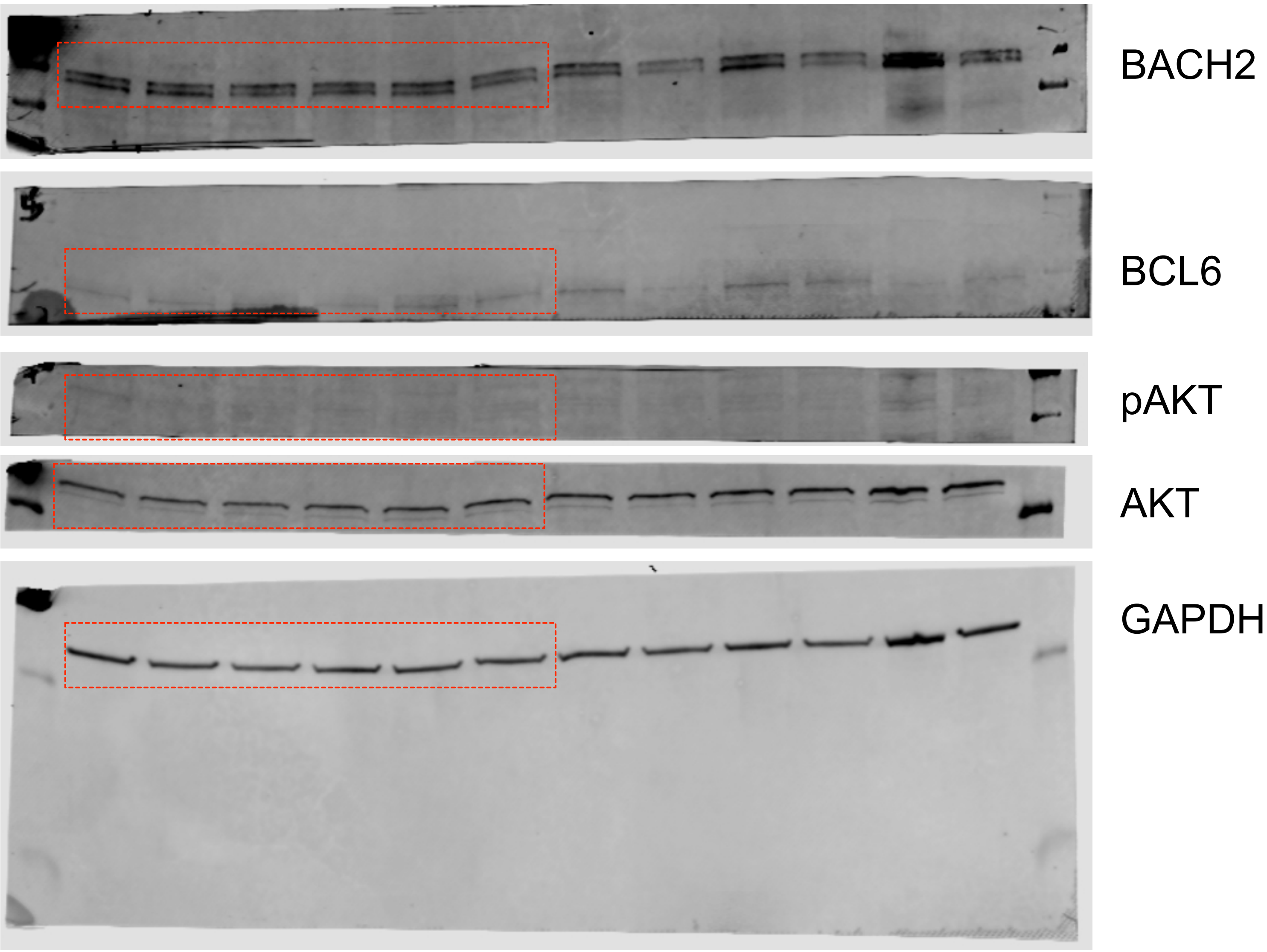

Original Blots Figure S3d

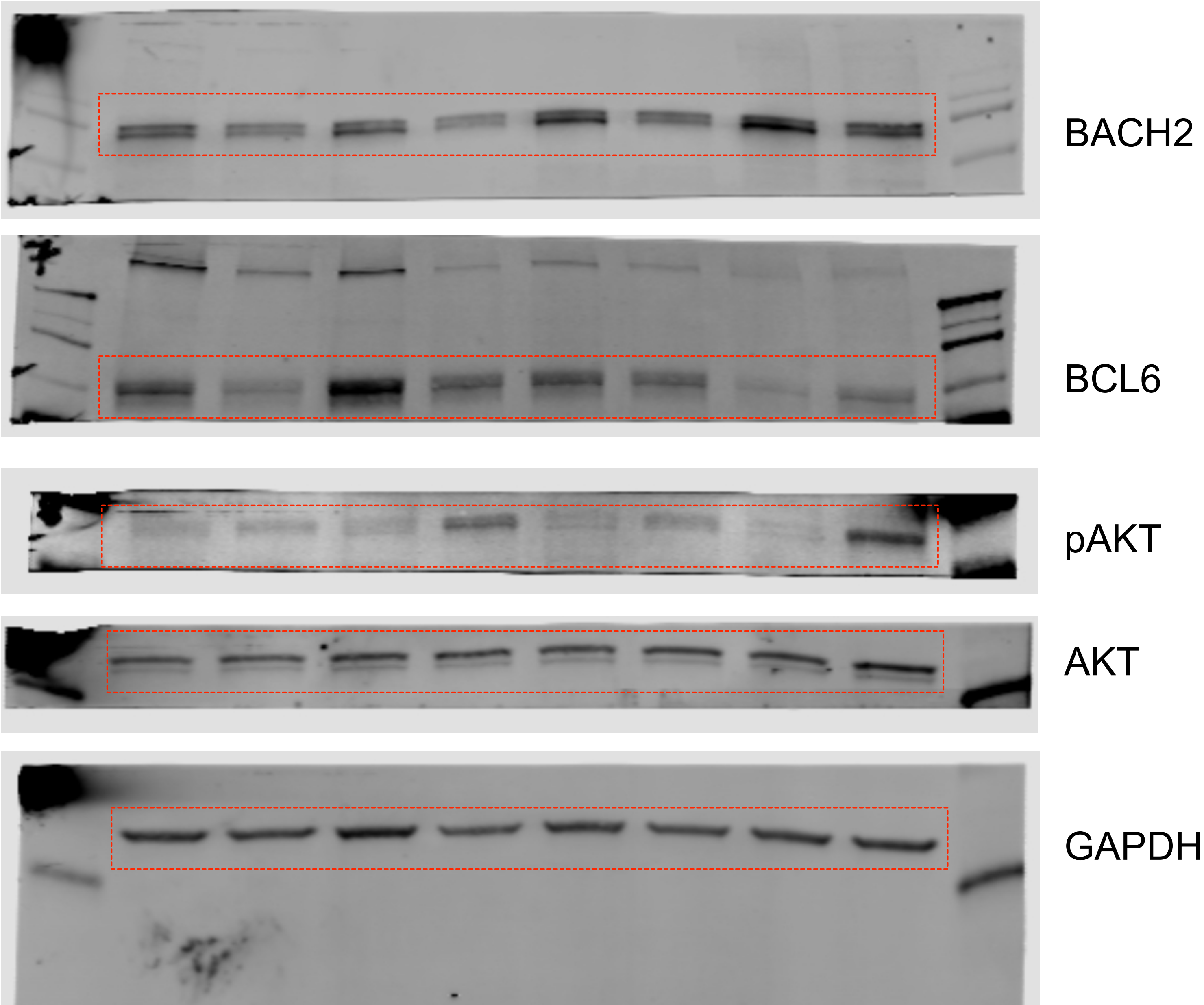

Supplement: Supplementary file 8 — Additional File 8: Figure S5. Original blots for Fig. 3b, c, S2, S3. Red boxes indicate the regions used in the respective Figures. [file 12885_2020_6650_MOESM8_ESM.pdf]
